# The Chromosomal Association of the Smc5/6 Complex Depends on Cohesion and Predicts the Level of Sister Chromatid Entanglement

**DOI:** 10.1371/journal.pgen.1004680

**Published:** 2014-10-16

**Authors:** Kristian Jeppsson, Kristian K. Carlborg, Ryuichiro Nakato, Davide G. Berta, Ingrid Lilienthal, Takaharu Kanno, Arne Lindqvist, Maartje C. Brink, Nico P. Dantuma, Yuki Katou, Katsuhiko Shirahige, Camilla Sjögren

**Affiliations:** 1 Karolinska Institutet, Department of Cell and Molecular Biology, Stockholm, Sweden; 2 University of Tokyo, Institute of Molecular and Cellular Biosciences, Laboratory of Genome Structure and Function Center for Epigenetic Disease, Bunkyo-ku, Tokyo, Japan; National Cancer Institute, United States of America

## Abstract

The cohesin complex, which is essential for sister chromatid cohesion and chromosome segregation, also inhibits resolution of sister chromatid intertwinings (SCIs) by the topoisomerase Top2. The cohesin-related Smc5/6 complex (Smc5/6) instead accumulates on chromosomes after Top2 inactivation, known to lead to a buildup of unresolved SCIs. This suggests that cohesin can influence the chromosomal association of Smc5/6 via its role in SCI protection. Using high-resolution ChIP-sequencing, we show that the localization of budding yeast Smc5/6 to duplicated chromosomes indeed depends on sister chromatid cohesion in wild-type and *top2-4* cells. Smc5/6 is found to be enriched at cohesin binding sites in the centromere-proximal regions in both cell types, but also along chromosome arms when replication has occurred under Top2-inhibiting conditions. Reactivation of Top2 after replication causes Smc5/6 to dissociate from chromosome arms, supporting the assumption that Smc5/6 associates with a Top2 substrate. It is also demonstrated that the amount of Smc5/6 on chromosomes positively correlates with the level of missegregation in *top2-4*, and that Smc5/6 promotes segregation of short chromosomes in the mutant. Altogether, this shows that the chromosomal localization of Smc5/6 predicts the presence of the chromatid segregation-inhibiting entities which accumulate in *top2-4* mutated cells. These are most likely SCIs, and our results thus indicate that, at least when Top2 is inhibited, Smc5/6 facilitates their resolution.

## Author Summary

When cells divide, sister chromatids have to be segregated away from each other for the daughter cells to obtain a correct set of chromosomes. Using yeast as model organism, we have analyzed the function of the cohesin and the Smc5/6 complexes, which are essential for chromosome segregation. Cohesin is known to hold sister chromatid together until segregation occurs, and our results show that cohesin also controls Smc5/6, which is found to associate to linked chromatids specifically. In line with this, our analysis points to that the chromosomal localization of Smc5/6 is an indicator of the level of entanglement between sister chromatids. When Smc5/6 is non-functional, the resolution of these entanglements is shown to be inhibited, thereby preventing segregation of chromatids. Our results also indicate that DNA entanglements are maintained on chromosomes at specific sites until segregation. In summary, we uncover new functions for cohesin, in regulating when and where Smc5/6 binds to chromosomes, and for the Smc5/6 complex in facilitating the resolution of sister chromatid entanglements.

## Introduction

**Figure 1:**
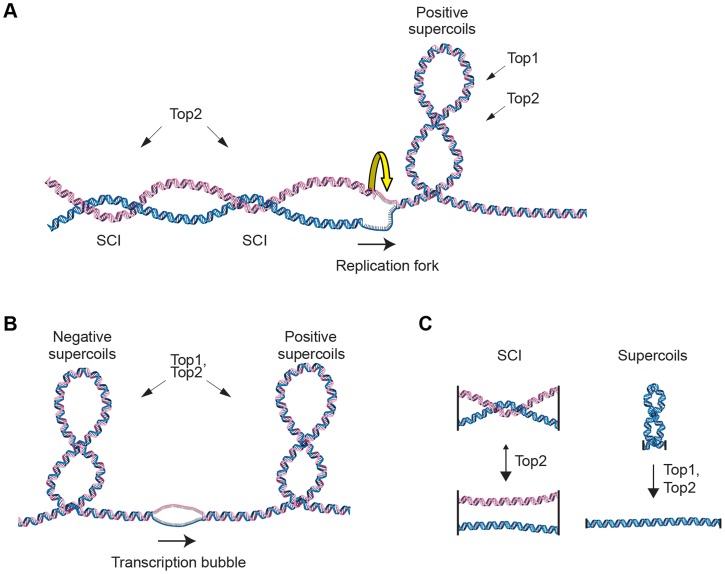
Schematic of DNA supercoiling, SCIs and topoisomerases. (**A**) Schematic figure of changes in DNA topology at the replication fork. Progressive separation of the parental DNA strands by the replication machinery leads to the accumulation of positive supercoils ahead of the replication fork. These can be resolved by both Top1 and Top2 topoisomerases in budding yeast. Sister chromatid intertwinings (SCIs) can be formed through rotation of the fork. Resolution of the SCIs by Top2 ensures full chromosome segregation during mitosis. (**B**) Schematic figure of changes in DNA topology due to transcription, positive supercoils accumulate ahead of the transcription machinery, while negative ones are found behind. (**C**) Top2 acts on SCIs, while both Top1 and Top2 can resolve supercoils.

In order to maintain chromosome stability, cells need to overcome topological problems caused by the structure of the DNA molecule. One example of such topological problem is DNA supercoiling induced by replication or transcription. Another is sister chromatid intertwinings (SCIs), which is the wrapping of chromatids around each other (Figure 1A and B). If not resolved by topoisomerases, supercoiling inhibits transcription and replication, and SCIs block chromosome segregation. While both type I and type II topoisomerases can resolve supercoils by making transient DNA breaks, the type II variant, called Top2 in the budding yeast *Saccharomyces cerevisiae* (*S. cerevisiae*), is main responsible for the resolution of SCIs (Figure 1C). [1]–[3]. If Top2 is rendered non-functional before anaphase, chromosome segregation with unresolved SCIs leads to DNA breakage and cell death [4]–[6]. In addition to presenting an obstacle for segregation, sister chromatid tethering by SCIs has been proposed to contribute to proper segregation by counteracting the force of the mitotic spindle, thereby facilitating chromosome alignment during metaphase [7]. The idea of such a positive function for SCIs was, however, challenged when the cohesin protein Scc1 (also known as Mcd1) was shown to be essential for sister chromatid cohesion [8], [9].

The cohesin complex, with a core consisting of Smc1, Smc3, Scc1 and Scc3, is a so-called Structural Maintenance of Chromosomes (SMC) protein complex. In addition to the four core subunits, the Pds5 protein associates to the complex via Scc1 [10], [11]. When either of the subunits is non-functional, sister chromatids are not held together, and chromosome alignment and segregation fail. Cohesin is loaded onto chromosomes before replication, and localizes to intergenic regions between genes that are transcribed in a convergent manner in *S. cerevisiae* [9], [12]. Several observations indicate that transcription drives the translocation of cohesin to these regions after initial loading by the Scc2/4 complex at centromeres and other, so far mostly undefined, chromosome arm sites [12]–[14]. In addition to loading, cohesin has to be modified in order to establish cohesion. A key regulator of this process is the acetyltransferase Eco1 (also called Ctf7) [15], [16], which acetylates Smc3 and thereby prevents the Pds5-associated protein Wpl1 (also called Rad61), to destabilize the cohesin-chromosome interaction [17]–[19]. In the absence of Wpl1, Eco1 becomes largely dispensable for cohesion establishment [18], [20], [21]. At anaphase, chromosome segregation is made possible by removal of cohesin from chromosomes by separase, a protease which cleaves the Scc1 subunit [22]. In higher eukaryotes, the proteolytic cleavage is preceded by a specific dissociation of cohesin along chromosome arms in prophase, leaving cohesin only in the centromeric region until anaphase [23], [24]. The identification of cohesin as the main constituent of chromatid cohesion provided an explanation of how sister chromatid cohesion could be maintained without the risk of chromosome breakage, which is inevitably linked to cohesion created by SCIs. A more recent study by Farcas *et al.* shows, however, that cohesin protects SCIs from resolution by Top2 on circular mini-chromosomes [25], suggesting SCIs could contribute to cohesion.

Intriguingly, the cohesin-related Smc5/6 complex (Smc5/6) has also been connected to Top2 function [26]. Smc5/6 consists of Smc5, Smc6 and six non-SMC proteins (Nse1, Mms21, and Nse3-6), and is best known for its function in DNA repair and recombination (reviewed in [27]). The complex is recruited to DNA breaks in a process dependent on Mre11, and central repair factor which accumulates early at the site of damage [28]. When Smc5/6 is non-functional, unresolved recombination intermediates accumulate between sister chromatids in the repetitive ribosomal DNA in unchallenged cells, and during S-phase repair of induced DNA damage [29]–[32]. Since DNA repair in the absence of proper Smc5/6 function is taken to a step that inactivates the DNA damage checkpoint, the unresolved DNA links will inhibit the subsequent segregation event. Also in meiosis, repair of DNA breaks without Smc5/6 leads to similar formation of unresolved recombination intermediates with following segregation failure [33]–[35]. In addition to this, Smc5/6 appears to have non-repair functions. In *S. cerevisiae*, Smc5/6 has been proposed to function in replication termination [36], and removal of replication-induced supercoiling [26]. Moreover, Smc6 has been reported to allow full removal of cohesin at anaphase when Top2 function is partially compromised in the fission yeast *Schizosaccharomyces pombe* (*S. pombe*) [37]. Concerning the chromosomal association of Smc5/6 in the absence of DNA damage, it is independent of Mre11, but requires the replication process as such, and increases after inactivation of the temperature-sensitive *top2-4* allele in *S. cerevisiae* [26], [28]. This opens for the possibility that the chromosomal association is triggered by the presence of SCIs, or another feature which accumulates in *top2-4*. Regardless if the complex is recruited to SCIs or recombination structures in the absence of DNA damage, its chromosomal association should require that sister chromatids are in close proximity to one another. This predicts that the levels of Smc5/6 present on the replicated genome should decrease in the absence of cohesion, which leads to a separation of chromatids before anaphase. However, our earlier ChIP-on-chip analysis (Chromatin immunoprecipitation (ChIP), combined with analysis on microarrays) of FLAG-tagged Smc6 indicated that the chromosome binding of Smc5/6 changed into more numerous, but narrower, binding sites in *scc1-73* cells [28]. The finding that the chromosomal association of Smc5/6 was not reduced in the absence of cohesin argued against it being triggered by a structure which requires the proximity of sister chromatids. In contrast, the *scc2-4* mutation, which inhibits cohesin loading, was shown to reduce the levels of chromosome-bound Smc6. This, together with the aberrant binding pattern of Smc6 in *scc1-73* cells, made it difficult to draw a definite conclusion on how cohesin influences the chromosomal association of Smc5/6. Using ChIP-sequencing (ChIP-seq, ChIP combined with DNA sequencing), together with ChIP-qPCR (ChIP combined with quantitative PCR) and *in situ* immunofluorescence, we now show that Smc5/6 chromosome binding is cohesin-dependent. The majority of the chromosome-bound Smc5/6 also co-localizes with cohesin in the vicinity of centromeres, and specifically accumulates along chromosome arms after Top2 inactivation. Evidence is provided that this accumulation is independent of recombination, DNA breaks and fork stalling. Our results also show that the amount of chromosome-bound Smc5/6 predicts the level of missegregation in *top2-4* cells, and that the complex promotes the segregation of short chromosomes in the mutant. Altogether, the presented data suggests that Smc5/6 indicates the presence of SCIs in the duplicated genome, and that the complex promotes their resolution, at least when Top2 is inhibited.

## Results

### The chromosomal association of Smc5/6 requires the cohesin complex

**Figure 2:**
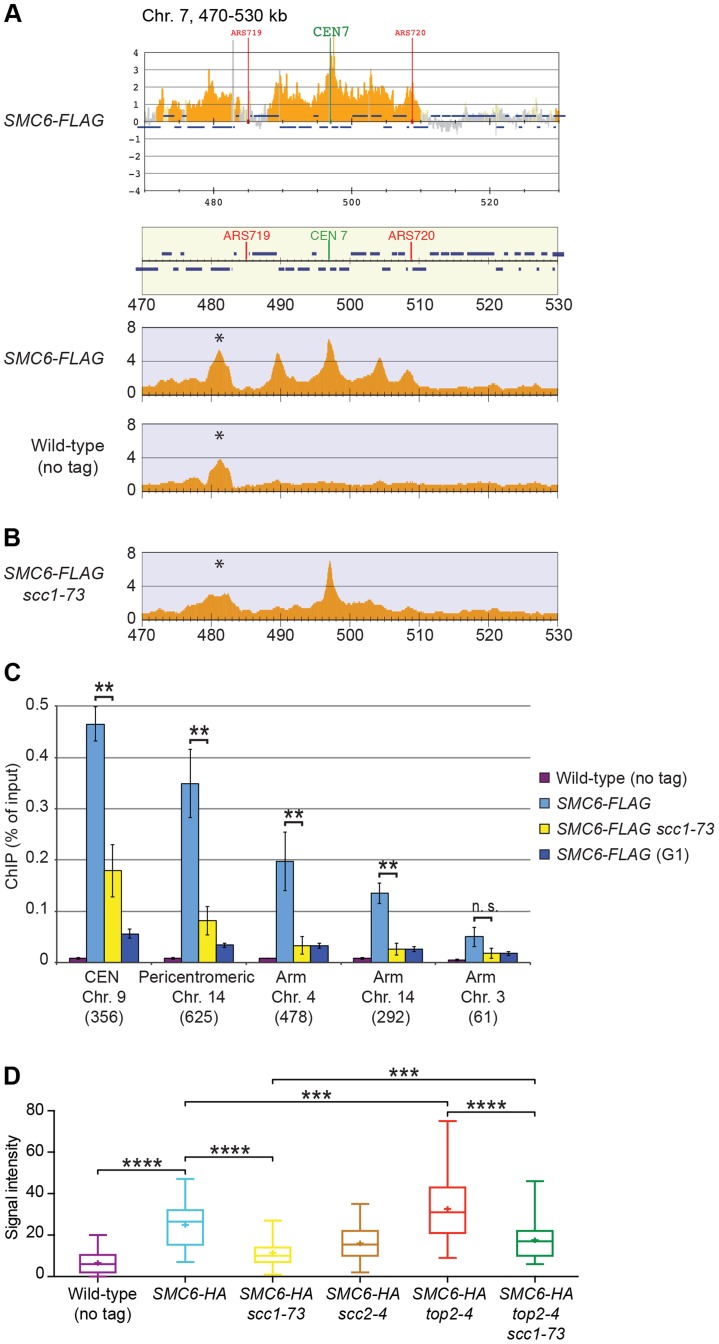
The chromosomal association of Smc6 depends on cohesin. (**A**) Comparison of ChIP-on-chip and ChIP-seq. Chromosomal localization of Smc6-FLAG as determined by ChIP-on-chip (upper panel) and ChIP-seq (middle panel). The lowest panel shows a ChIP-seq map from a control experiment performed on cells lacking FLAG-tagged proteins. (**B**) Chromosomal association of Smc6-FLAG in G2/M-arrested *scc1-73* cells as determined by ChIP-seq. In both (A) and (B), cells were arrested in G2/M after a synchronous S-phase at 35°C before sample preparation. The asterisk (*) denotes a false positive peak. The Y-axis shows fold enrichment of ChIP/whole cell extract (WCE) in log scale for ChIP-on-chip and in linear scale for ChIP-seq, while the X-axis shows chromosomal positions. Blue horizontal bars in the panel describing the genomic region denote open reading frames, while red and green vertical lines denote replication origins (ARS) and centromeres (CEN), respectively. (**C**) Chromosomal association of Smc6-FLAG at selected chromosomal positions in indicated strains as determined by ChIP-qPCR. Cells were arrested in G2/M after a synchronous S-phase at 35°C, or after G1 arrest (G1), before sample preparation. Chromosome number and distance from the left telomere of each analyzed position is indicated below the corresponding group of bars. Y-axis displays the amount of DNA in ChIP fraction in relation to input. Each bar represents the mean of three independent experiments with standard deviations indicated. Statistical analysis was performed using the Student's two-tailed *t*-test. *P*-values are illustrated in the figures as follows: n. s. = not significant (*p*>0.05); * = significant (*p*<0.05); ** = significant (*p*<0.01); *** = significant (*p*<0.001). Maps displaying Smc6 enrichment at the corresponding sites as determined by ChIP-seq are displayed in Figure S2. (**D**) Chromosomal association of Smc6-HA as determined by immunofluorescence of chromosome spreads. Indicated *SMC6-3HA*-expressing cells were arrested in G2/M after a synchronous S-phase at 35°C. Samples were collected and chromosome spreads were prepared and stained to detect the HA-epitope tag on Smc6. Box whisker plots representing the signal intensity in arbitrary units (AU) from quantifications of at least 50 cells for each sample are shown. Statistical analysis was done using a two-tailed *t*-test with Welch's correction. *P*-values are as follows: Wild-type (no tag) vs. *SMC6-3HA*, *p*<0.0001 (****); *SMC6-3HA* vs. *scc1-73 SMC6-3HA*, *p*<0.0001 (****); *SMC6-3HA* vs. *top2-4 SMC6-3HA*, *p* = 0.0006 (***);*scc1-73 SMC6-3HA* vs. *top2-4 scc1-73 SMC6-3HA*, *p* = 0.0002 (***); *top2-4 SMC6-3HA* vs. *top2-4 scc1-73 SMC6-3HA*, *p*<0.0001 (****).

Triggered by the observations that Smc5/6 accumulates on chromosomes in *top2-4* mutants [26], and that cohesin is a protector of SCIs [25], we revisited the chromosomal association of *S. cerevisiae* Smc5/6 using ChIP-seq. This method is more quantitative than ChIP-on-chip, and provides more clearly defined binding sites (Figure 2A). The difference is likely caused by the requirement of additional amplification of the immunoprecipitated DNA in ChIP-on-chip, which increases the risk of false positive signals due to the preferential augmentation of certain DNA molecules. Moreover, 50 base pairs (bp) reads are mapped to a reference genome in ChIP-seq, while the amplified material is hybridized to 25 bases long oligonucleotides, each representing a specific genomic sequence, in ChIP-on-chip. The short length of the oligonucleotides, and the requirement for hybridization as such (the efficiency of which varies from oligonucleotide to oligonucleotide), makes the ChIP-on-chip method less accurate.

In contrast to previous results, the ChIP-seq analysis showed that the levels of Smc6 found on chromosomes were markedly reduced in the *scc1-73* mutant after an S-phase at restrictive temperature (Figure 2B). Western blot analysis confirmed that this reduction was not due to a general down-regulation of Smc6-FLAG protein levels in the mutant (Figure S1A). At core centromeres the signal remained high, but at all other specific Smc6 binding sites it was abolished (Figure 2B). The reduction of Smc6 was confirmed by ChIP-qPCR in the *scc1-73* mutant (Figures 2C and S2). At arm loci, the amount of Smc6 was reduced to levels similar to those in G1-arrested wild-type cells, reflecting the background level before the complex has associated with chromosomes. The Smc6 signal around centromeres was also significantly reduced but remained at up to one third of the wild-type level (Figure 2C). Thus, the ChIP-qPCR results show that the ChIP-seq data is quantitatively accurate, apart from at core centromeres where the signal is overestimated in ChIP-seq, when few other binding sites are present.

Even though this indicates that Smc5/6 is largely absent from chromosomes in the cohesin mutant, it is possible that the reduction only reflects the spreading of the complex to an even distribution over the chromosomes. Such redistribution would make it undetectable by ChIP-seq and lead to a reduction in the ChIP-qPCR signal. To test if this was the case, immunofluorescence (IF) was utilized to detect the association of HA-tagged Smc6 on chromosome spreads. In *scc1-73* cells, the fluorescence signal was reduced towards the levels detected in untagged cells (Figure 2D). As for Smc6-Flag, the signal reduction was not due to lower levels of the Smc6-HA protein (Figure S1B), showing that the chromosomal association, and not only positioning, of Smc5/6 requires a functional cohesin complex.

### The chromosomal association of Smc5/6 requires sister chromatid cohesion

**Figure 3:**
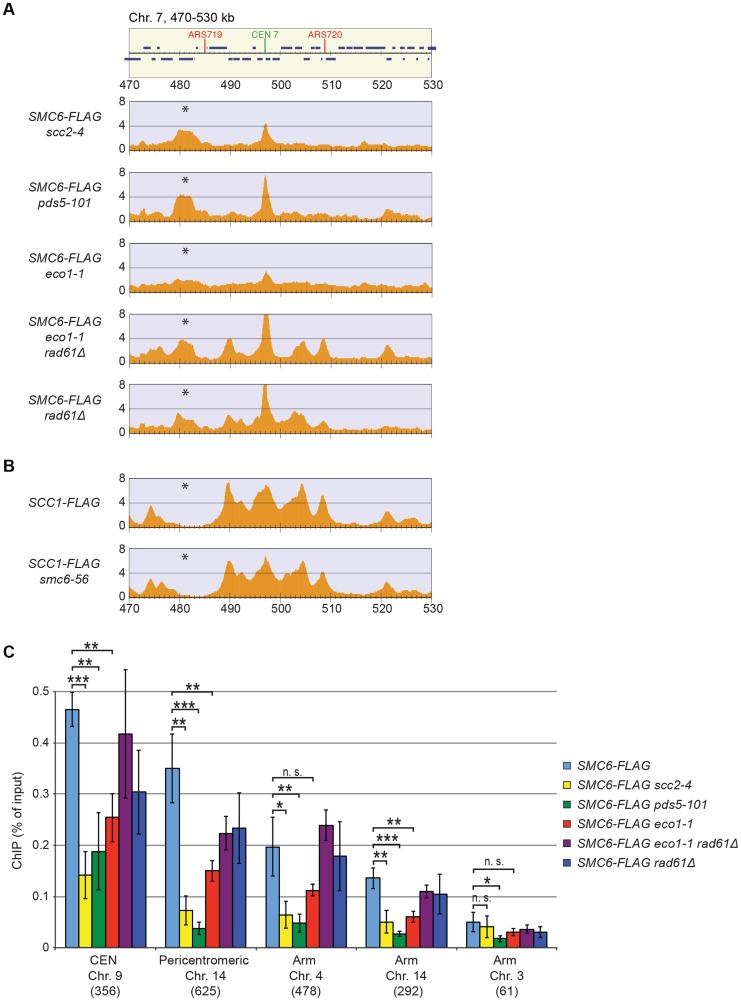
The chromosomal association of Smc5/6 depends on sister chromatid cohesion. (**A**–**C**) Chromosomal association of (**A** and **C**) Smc6-FLAG and (**B**) Scc1-FLAG in indicated strains as determined by (**A** and **B**) ChIP-seq and (**C**) ChIP-qPCR. All cells were arrested in G2/M after a synchronous S-phase at 35°C before sample preparation. Note that the false positive signal in denoted (*) is mostly absent in *SCC1-FLAG* cells. Figure details and statistical analysis are described in Figure 2. In (C), results for Smc6-FLAG in wild-type cells are identical to those displayed in Figure 2C, and shown for comparison.

The reduction of Smc6 binding in *scc1-73* mutants indicates that Smc5/6 requires sister chromatid cohesion to associate with chromosomes. To test this further, Smc6 localization was analyzed in other cohesion-disrupting mutants. First, ChIP-seq and ChIP-qPCR analysis confirmed the earlier result that Smc5/6 requires the cohesin-loading protein Scc2 for chromosomal association (Figure 3A and C) [28]. The reduction of Smc6 binding in *scc1-73* and *scc2-4* mutants as measured by ChIP-qPCR was similar (Figures 2C and 3C), and the reason for the difference previously seen by ChIP-on-chip remains unknown [28]. We also found that binding of Smc6 was prevented in the temperature sensitive *pds5-101* mutant (Figure 3A and C). ChIP-seq was also performed on Smc6-FLAG in *eco1-1* cells, in which formation of sister chromatid cohesion is inhibited even though cohesin remains bound to the chromatids [15], [16]. The reduction of Smc6 binding (Figure 3A and C) in this mutant therefore shows that the chromosomal association of Smc5/6 requires cohesion, and not merely the presence of cohesin on chromosomes. This was further supported by the observation that Smc6 chromosome binding in *eco1-1* cells was increased by deletion of Wpl1 (Rad61) (Figure 3A and C), which restores cohesion [18], [20], [21]. On the other hand, the localization of cohesin remained unchanged in an *smc6-56* mutant after an S-phase at restrictive temperature, showing that although cohesin controls Smc5/6, the reverse is not true (Figure 3B). Altogether, this shows that sister chromatids have to be held together for Smc5/6 to bind to the duplicated genome.

### Smc5/6 accumulates at replicated cohesin binding sites

**Figure 4:**
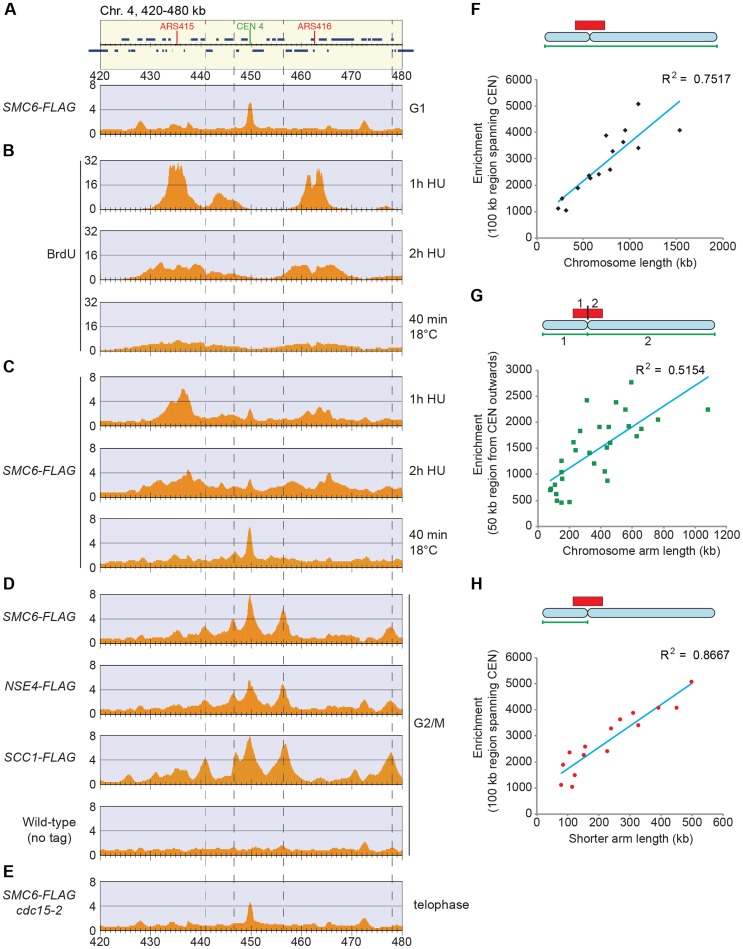
Smc5/6 is enriched on replicated chromosomes in between convergently transcribed genes close to centromeres, and the level of enrichment increases if the centromeres are distant from a chromosome end. (**A**–**C**) Chromosomal localization of Smc6-FLAG during G1 (**A**) and S-phase (**C**), or bromodeoxyuridine (BrdU) incorporation (**B**), as determined by ChIP-seq. In (B and C), cells were arrested in G1 and subsequently released into media containing BrdU in the presence of hydroxyurea (HU), or at 18°C as indicated. Samples were collected in HU and 1 and 2 hours after the release. ChIP-seq analysis was performed using anti-FLAG (**A** and **C**), or anti-BrdU antibodies (**B**). (**D**) Chromosomal localization of Smc6-FLAG, Nse4-FLAG and Scc1-FLAG in G2/M-arrested cells. Samples were collected in a nocodazole-induced G2/M-arrest after a synchronous S-phase at 35°C. (**E**) Chromosomal localization of Smc6-FLAG cells arrested in telophase due to non-functional Cdc15 mitotic exit network kinase. Samples were prepared as in (D) but released from G1-arrest in nocodazole-free medium. Panel details as in Figure 2 with the addition of vertical dashed lines showing that Smc6-FLAG and Nse4-FLAG co-localize with Scc1 in between convergently oriented genes in the G2/M-arrest. (**F**–**H**) Scatterplots comparing Smc6-FLAG enrichment with full chromosome length (**F**), chromosome arm length (**G**), or the distance from the centromere to the nearest telomere (i. e. the length of the shorter chromosome arm) (**H**). Enrichment values represent Smc6-FLAG enrichment in 100 kb regions spanning the centromeres (**F** and **H**) or in 50 kb regions to the left or right of the centromere (**G**). Enrichment values from ChIP-seq performed on a control strain lacking tagged proteins were subtracted for each region (see Figure S4). Correlation values are indicated in each graph. Chromosome 12 was excluded from the scatterplots due to the unknown length of the rDNA present on that chromosome.

To take full advantage of the higher resolution obtained by ChIP-seq as compared to ChIP-on-chip, we reinvestigated the chromosomal association of Smc5/6 during the cell cycle. This confirmed that the complex is mostly absent from chromosomes in G1. Similarly to the Smc6 binding pattern in G2/M-arrested *scc1-73* cells, ChIP-seq also revealed an association to the core centromeres in this cell cycle phase (Figure 4A), but ChIP-qPCR analysis showed that the levels are low compared to the binding in G2/M-arrested cells (Figure 2C). As shown before, Smc5/6 is detected at stalled forks in cells arrested in early S-phase by the addition of hydroxyurea (HU) [38], which is a binding pattern that differs from the distribution found after completion of replication (Figure 4B–D). This could indicate that the Smc5/6 is associated with the fork and follows fork progression, and to test this, Smc6 binding was analyzed in cells progressing through S-phase at 18°C. This condition is generally applied to slow down replication and to improve cell cycle synchronization. As in the HU-arrested cells, Smc6 displayed a different binding pattern as compared to in G2/M, but the signals were less well defined, likely due to a lower level of synchronization (Figure 4B–D). Even though this left the question whether Smc5/6 follows the replication fork unanswered, it shows that the binding pattern detected in G2/M is not present in early S-phase.

In addition to this, the following new features were revealed. First, Smc5/6 is absent from chromosomes in telophase cells, arrested through inactivation of the mitotic exit network kinase Cdc15 (Figure 4E), well in line with the dependency on cohesin, which is removed from chromosomes at anaphase onset. Second, robust Smc5/6 binding sites are concentrated around the centromeres in G2/M-arrested cells, and all of these sites are found between convergently transcribed genes and co-localizes with cohesin (Figures 4D and S3). A third new feature of Smc6 chromosomal association was detected when comparing the level of association with the length of each chromosome. Earlier analysis showed that Smc5/6 enrichment per chromosome increased with its length [26], [28]. Due to the new observation that strong Smc5/6 chromosome interaction sites clusters around centromeres, this analysis was repeated focusing on this region. Smc6 enrichment was calculated in a 100 kb region spanning the centromere (Figure S4), and when compared to chromosome length, a positive correlation was confirmed (Figure 4F). In our earlier analysis, we suggested that this binding pattern reflects that SCIs can swivel off chromosome ends [26]. If so, enrichment on each side of the centromere should also correlate to the length of the corresponding chromosome arm. This is because kinetochores are re-attached to microtubules directly after their replication, which should confine SCIs to each individual arm [39]. However, the correlation between Smc6 enrichment in a 50 kb region on either side of the centromere and the length of corresponding chromosome arm is low, arguing against such an interpretation (Figure 4G). On the other hand, the levels of Smc6 in the entire 100 kb region showed a stronger correlation with the distance to the closest telomere, i. e. the length of the shortest chromosome arm (Figure 4H). This shows that the further away from a chromosome end a centromere is positioned, the more Smc5/6 will accumulate in its vicinity.

### Top2 inactivation leads to cohesin-dependent accumulation of Smc5/6 along chromosome arms

**Figure 5:**
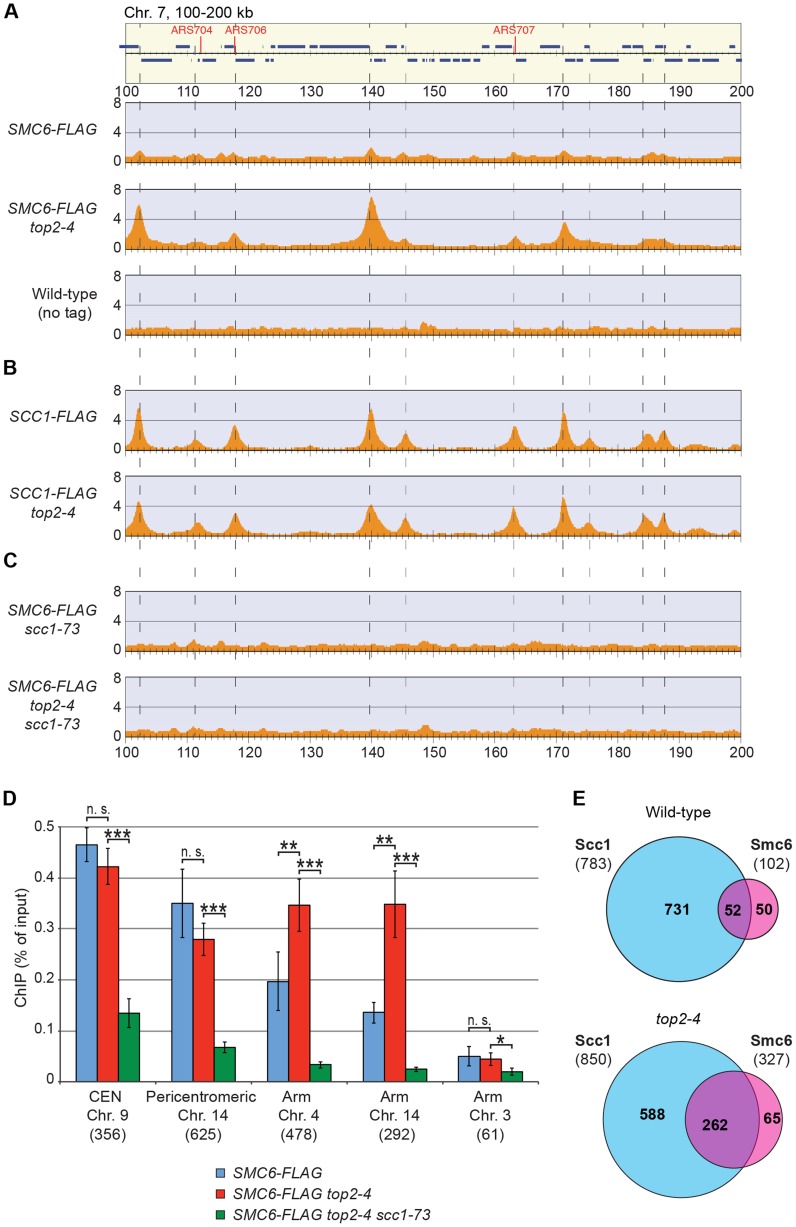
Smc5/6 accumulates on chromosomes after Top2 inhibition, and co-localizes with cohesin in both wild-type and *top2-4* cells. (**A–D**) Chromosomal association of (**A**, **C** and **D**) Smc6-FLAG and (**B**) Scc1-FLAG in indicated strains as determined by (**A–C**) ChIP-seq and (**D**) ChIP-qPCR. All cells were arrested in G2/M after a synchronous S-phase at 35°C before sample preparation. The ChIP-seq maps displays a chromosomal region spanning 100–200 kb from the left telomere of chromosome 7. Panel details and statistical analysis are described in Figure 2. In (D), results for Smc6-FLAG in wild-type cells are identical to those displayed in Figure 2C, and shown for comparison. (**E**) Overlap of Smc6 and Scc1 binding sites on chromosome arms. For annotation of Smc6 and Scc1 binding sites, see Material and Methods.

Having determined that the chromosomal localization of Smc5/6 depends on cohesin and cohesion, the chromosomal binding pattern in *top2-4* cells was determined using ChIP-seq and ChIP-qPCR. These analyses showed that Smc6 binding around centromeres in *top2-4* was not significantly changed as compared to wild-type cells. However, along chromosome arms, Smc6 was strongly enriched at specific sites (Figures 5A and S3). Such an accumulation of Smc6 was also detected after depletion of Top2 by induced protein degradation, showing that it is was not specific effect of the *top2-4* allele (Figure S5). In contrast to *top2-4* cells, however, the Smc6 signal was increased at core centromere 9 after Top2 depletion, opening for a functional difference at these sites. The reason for this difference is unknown and here we focus on the increase along chromosome arms, which is common to both conditions. Similar to the binding sites in the pericentromeric region, the new binding sites were mainly found in intergenic regions between convergently oriented genes and co-localized with cohesin in *top2-4* cells (Figure 5A, B and E). The binding pattern of Scc1, on the other hand, remained unaltered in *top2-4* cells, showing that the change in Smc6 association does not reflect alterations in cohesin's chromosomal localization (Figure 5B). Moreover, the levels of chromosome-bound Smc6, as determined by ChIP-seq and IF, were reduced not only in *scc1-73* cells, but also in *top2-4 scc1-73* cells after an S-phase under restrictive conditions (Figures 5C and 2D). This reduction was confirmed by ChIP-qPCR (Figure 5D), and as in *scc1-73* cells, it was not due to lower Smc6 protein levels in *top2-4 scc1-73* cells (Figure S1A and B). This suggests that the chromosomal binding of Smc5/6 in wild-type and *top2-4* is due to the same underlying cohesin-dependent mechanism. However, even though the IF analysis showed that the level of chromosome-bound Smc6 was lower in *top2-4 scc1-73* than in *top2-4* cells, the signal was significantly stronger than in the *scc1-73* single mutant (Figure 2D). This, together with the ChIP results, indicates that some Smc5/6 remains on chromosomes in *top2-4 scc1-73*, but distributes differently from cells with functional cohesin.

### The increase of chromosome-bound Smc5/6 in *top2-4* cells requires passage through S-phase at restrictive temperature

**Figure 6:**
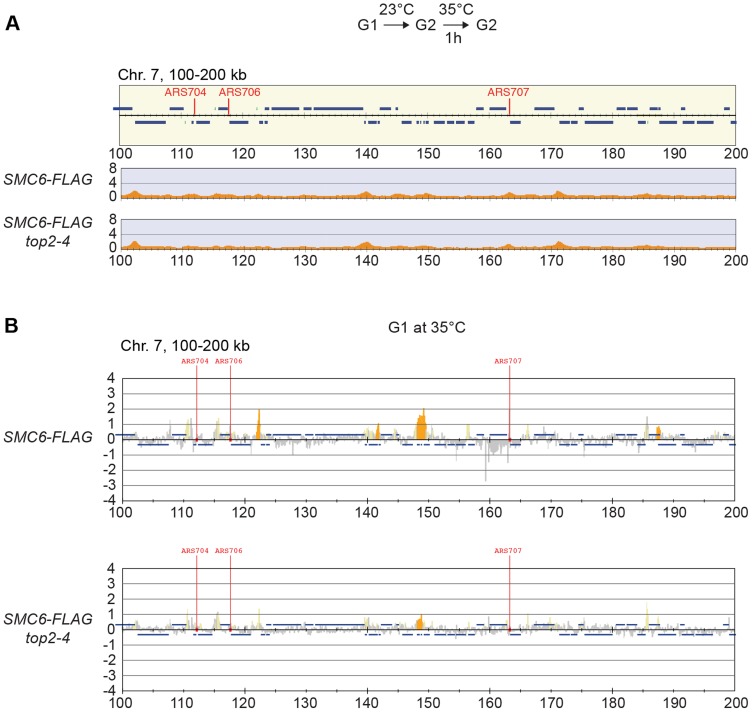
The increase of chromosome-bound Smc5/6 in *top2-4* cells requires passage through S-phase at restrictive temperature. (**A**) Chromosome arm association of Smc6-FLAG in G2/M-arrested *top2-4* cells as determined by ChIP-seq. Samples were collected after 1 hour temperature increase to 35°C under maintained arrest. Note the lack of accumulation of Smc6-FLAG in this region, as compared if Top2 is inactivated during replication (Figure 5A). (**B**) Analysis of Smc6-FLAG in G1-arrested wild-type and *top2-4* cells by ChIP-on-chip. Samples were collected after a 30 minutes at 35°C in a sustained G1-arrest. A region spanning 100–200 kb from the left telomere of chromosome 7 is shown. Panel details are described in Figure 2.

Knowing that Top2 is needed for removal of transcription-induced supercoils [2], [40], the accumulation of Smc5/6 in *top2-4* could be controlled by transcription alone. To investigate this, ChIP-seq and ChIP-on-chip analysis was performed after 1 hour of Top2 inactivation in G2/M-arrested cells, or after 30 minutes inactivation in a G1-arrest. The results revealed that without passage through S-phase at restrictive conditions, there was no alteration in Smc6 chromosomal interaction pattern as compared to the wild-type binding pattern (Figure 6A and B). This shows that like in wild-type cells, the chromosomal positioning of Smc5/6 is set under replication in the *top2-4* mutant.

### The chromosomal association of Smc5/6 is independent of recombination, DNA breaks, and replication fork stalling

**Figure 7:**
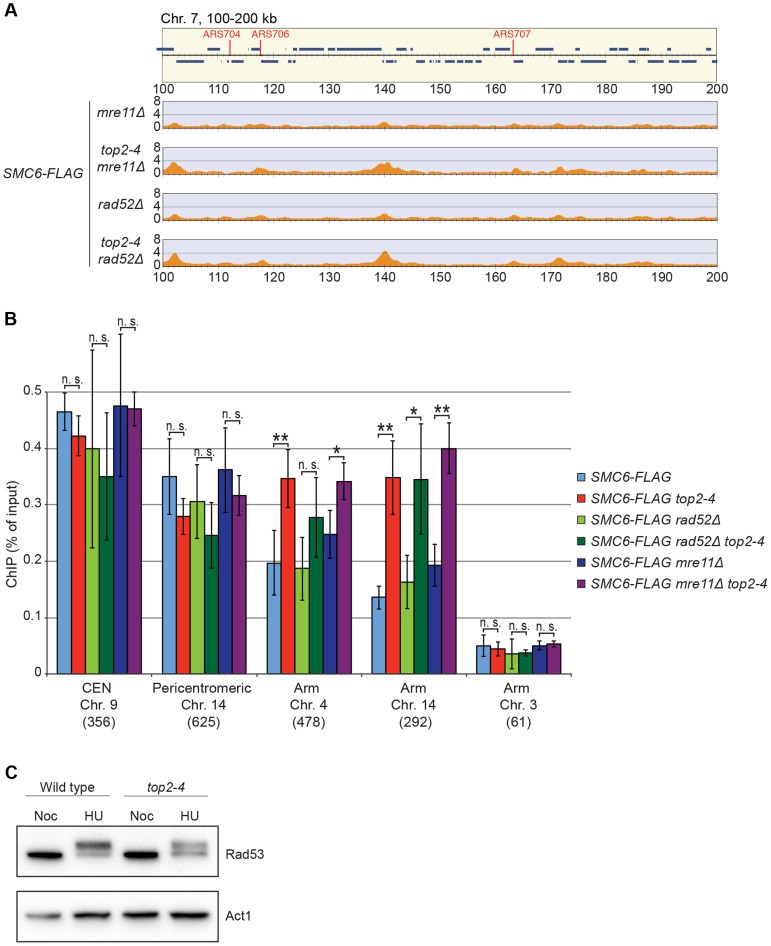
The chromosomal association of Smc6 does not depend on DSB formation or recombination. (**A** and **B**) Chromosome arm association of Smc6-FLAG in G2/M-arrested *mre11Δ*, *top2-4 mre11Δ*, *rad52Δ*, *top2-4 rad52Δ* cells as indicated, determined by ChIP-seq (**A**) and ChIP-qPCR (**B**). Samples were collected in G2/M after a synchronous S-phase at 35°C, nonpermissive for the *top2-4* allele. In (A), a region spanning 100–200 kb from the left telomere of chromosome 7 is shown. Panel details for (A) and (B) are described in the legend of Figure 2. (**C**) Western blot of Rad53 and actin in wild-type and *top2-4* cells arrested in S-phase by HU or in G2/M by nocodazole, as indicated.

It is well established that Smc5/6 is recruited to DNA double-strand breaks (DSBs) and facilitates resolution of recombination intermediates [28], [32], [41]. To test whether Smc5/6 chromosome association in *top2-4* cells was dependent on these structures, ChIP-seq and ChIP-qPCR analysis of Smc6 was performed on cells lacking *RAD52* or *MRE11*. Deletions of these genes inhibit recombination and Smc5/6 recruitment to DSBs, respectively [28], [42]. The results showed that Smc6 still accumulates on chromosomes when Top2 is inhibited in these mutants, demonstrating that the complex binds chromosomes independently of DNA breaks and recombination in *top2-4* cells (Figure 7A and B). This was further supported by western blot analysis of the Rad53 kinase, which is part of the damage cell cycle checkpoint and becomes phosphorylated upon DNA damage [43], [44]. This phosphorylation can be detected as a slower migrating form of Rad53, and this was readily observed after replication inhibition through the addition HU to both wild-type and *top2-4* cells (Figure 7C). However, after passage through S-phase in the restrictive temperature without addition of HU, no phosphorylation was detected. This indicates that no DNA damage accumulates upon inhibition of Top2 during S-phase.

**Figure 8:**
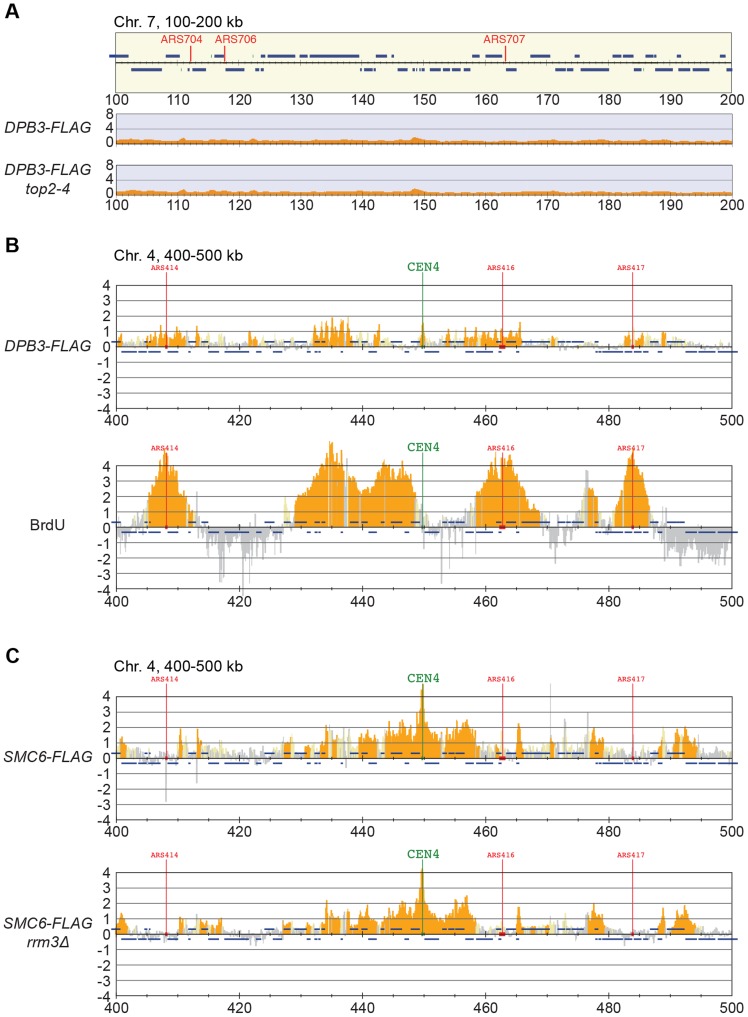
The chromosomal association of Smc6 does not depend on replication fork stalling. (**A**) ChIP-seq analysis of FLAG-tagged DNA polymerase II subunit Dpb3 in G2/M-arrested wild-type and *top2-4* cells. Compare to the enrichment of Smc6-FLAG seen at specific loci in this region in Figure 5A (**B**) ChIP-on-chip analysis of chromosome association of FLAG-tagged Dpb3 (upper panel) and BrdU-incorporation (lower panel) in HU-arrested S-phase cells. (**C**) Chromosome arm association of Smc6-FLAG in G2/M-arrested *rrm3Δ* cells. G2/M-samples were collected after a synchronous S-phase at 35°C, nonpermissive for the *top2-4* allele (**A**). For analysis in S-phase, cells were arrested in G1 and subsequently released into media containing BrdU and HU. Samples were collected 1 hour after the release (**B**). Analysis was performed using anti-FLAG (**A**, upper panel in **B**, and **C**), or anti-BrdU antibodies (lower panel in **B**). A region spanning 100–200 kb from the left telomere of chromosome 7 is shown, panel details are described in Figure 2.

**Figure 9:**
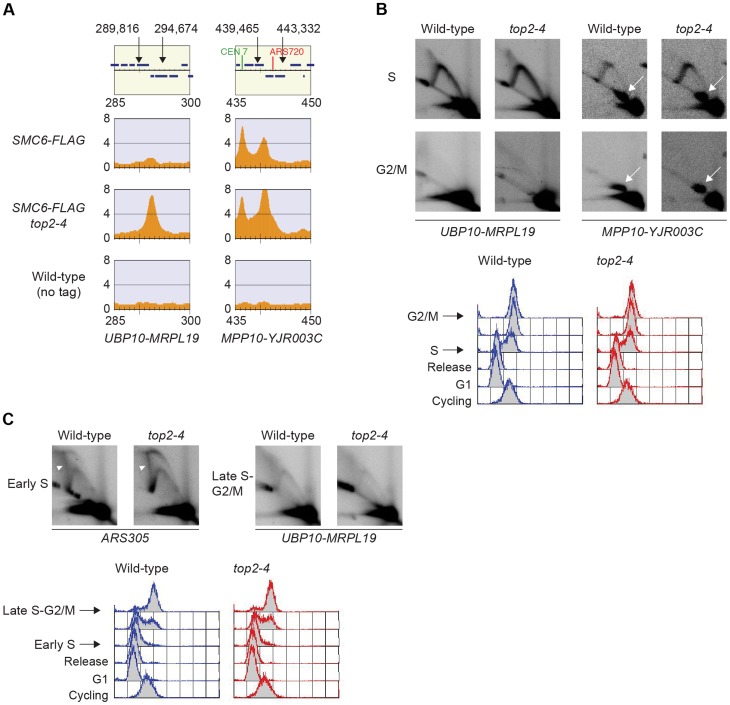
Chromosomal regions where Smc5/6 accumulates after Top2 inhibition show no sign of persistent replication or recombination intermediates. (**A**) Chromosomal localization of Smc6-FLAG as determined by ChIP-seq at two loci, *UBP10-MRPL19* and *MPP10-YJR003C*, showing abundant Smc6-FLAG binding in *top2-4*. The lowest panel shows a ChIP-seq map from a control experiment performed on cells lacking FLAG-tagged proteins. Panel details and cellular growth conditions are as described in the legend of Figure 2. In the top panel describing genomic features, arrows and chromosomal positions denote the restriction sites for *PstI*, used to produce the analyzed fragments. (**B**) Two-dimensional gel electrophoresis of *UBP10-MRPL19* (*left*) and *MPP10-YJR003C* (*right*) in wild-type and *top2-4* cells. Cell cycle progression monitored by fluorescence-activated cell sorting (FACS) is shown below and time-points of sample preparation are indicated. Membranes were first probed against *UBP10-MRPL19* (*left*), then stripped and re-probed against *MPP10-YJR003C* (*right*), leaving some residual signal from *UBP10-MRPL19* in the *MPP10-YJR003C* blots (white arrows). (**C**) Two-dimensional gel electrophoresis after DNA isolation using CTAB-extraction to preserve X-shaped molecules, of *ARS305* (*left*) and *UBP10-MRPL19* (*right*) in wild-type and *top2-4* cells. The *ARS305* containing fragment was produced by digestion using *EcoRI* and *HindIII*, and is a positive control for X-shaped molecule isolation (white arrowheads). Cell cycle progression monitored by FACS is shown below and indicates the time-points of sample preparation.

Smc5/6 is also known to associate to stalled replication forks [38], and Top2 has been shown to facilitate termination of replication [45]. It is therefore possible that Smc5/6 marks stalled forks that are still present in G2/M-arrested *top2-4* cells. To test this, the chromosomal localization of the DNA polymerase epsilon subunit Dpb3 was analyzed. This showed that even though Dpb3 was detected on S-phase chromosomes, it was not found in G2/M-arrested *top2-4* cells (Figure 8A and B). Moreover, Smc6 did not accumulate on chromosomes in a helicase *rrm3Δ* mutant, known to elicit replication fork stalling (Figure 8C) [46]. Finally, to assay directly if replication or recombination intermediates accumulate at Smc5/6 binding sites, two-dimensional gel electrophoresis was performed at two loci displaying abundant Smc5/6 binding in *top2-4* cells (Figure 9A). At both loci, replication intermediates were detected in S-phase in wild-type and *top2-4* cells, but not in G2/M-arrested cells, when Smc5/6 binding is most abundant (Figure 9B). This shows that the accumulation of Smc5/6 at these loci in *top2-4* mutant is not due to the presence of a DNA structure that can be detected by a standard two-dimensional gel electrophoresis assay. In addition, the *UPB10-MRPL19* locus was investigated using two-dimensional gel electrophoresis on DNA prepared using a CTAB-extraction method [47]. This method preserves specific X-shaped structures, which have been suggested to be hemicatenated sister chromatids, and in early S-phase, these could be detected at a positive control locus, *ARS305*, in wild-type and *top2-4* cells (Figure 9C). In the end of S-phase, no difference between wild-type and *top2-4* cells could be seen at the Smc5/6 binding sites. This shows that it is not an increase in hemicatenane-like structures that causes Smc5/6 to accumulate after Top2 inhibition. Altogether, this indicates that the Smc5/6 binding pattern detected in *top2-4* cells is independent of DNA breaks, recombination and replication fork stalling.

### Restoration of Top2 function in G2/M triggers the dissociation of Smc5/6 from chromosomes

**Figure 10:**
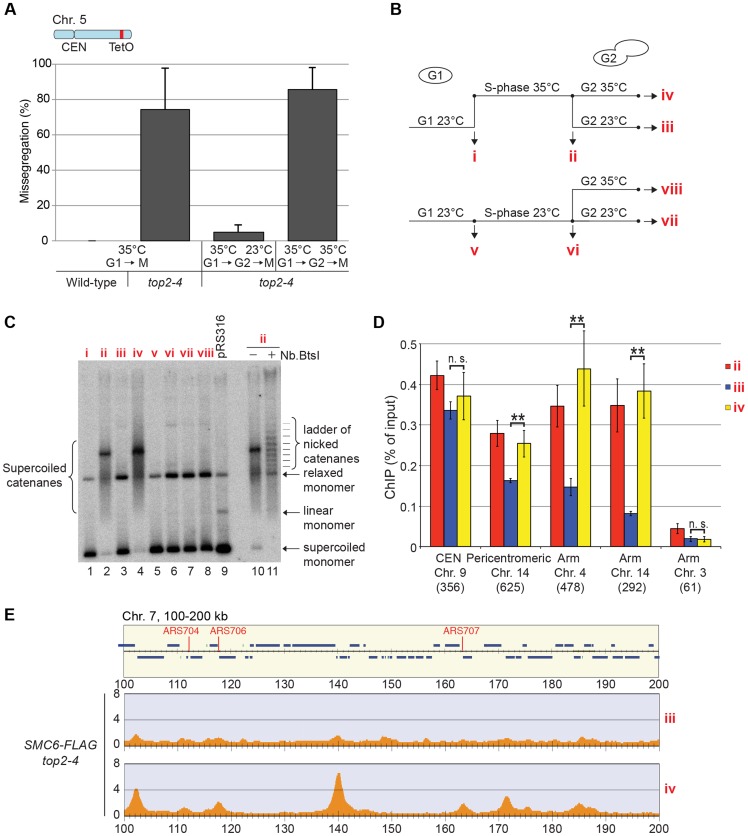
Restoration of Top2 function after replication removes Smc5/6 from chromosomes. (**A**) Segregation of a fluorescently labeled region located 72 kb from the right telomere of chromosome 5 in wild-type and *top2-4* cells. After G1-arrest at permissive temperature for *top2-4* (23°C), cells were incubated at the restrictive temperature 35°C during 30 minutes before release at the high temperature. Segregation was scored in next G1 without any intervening arrest, or after 1 hour in G2/M at 23°C or 35°C, as indicated. Missegregation was defined as events when chromosome 5 was absent from mother or daughter cell 70 minutes after the start of imaging, and only scored in cells in which spindle elongation had occurred. (**B**) Experimental setup in *C*–*E*. (**C**) Southern blot analysis of the plasmid pRS316, isolated from *top2-4* cells at time points indicated in (**B**). pRS316 is in supercoiled and relaxed monomeric forms in G1 (lane 1 and 5, compare to control plasmid isolated from bacteria, lane 9). After an S-phase at 35°C, pRS316 accumulates as a high molecular weight form (lane 2). This is supercoiled dimeric plasmids, as shown by its transformation into a ladder of relaxed dimeric molecules after treatment with the nicking enzyme *Nb.BtsI* (lane 10 and 11). Reactivation of Top2 through temperature down-shift resolves dimers into supercoiled and relaxed monomeric plasmids (lane 3), while maintained inactivation leaves the dimers unresolved (lane 4). After replication at 23°C, the plasmids remain in the monomeric forms, also after *top2-4* inactivation in G2/M (lanes 5–8). (**D**) Chromosomal association of Smc6-FLAG at selected chromosomal positions as determined by ChIP-qPCR. Chromosome number and distance from the left telomere of each position is indicated below the corresponding bar. Restoration of Top2 function by temperature down-shift in G2/M leads to dissociation of Smc6 from all tested binding sites except at the core centromere of chromosome 9. Results for Smc6-FLAG in *top2-4* cells are identical to those displayed in Figure 5D, and shown for comparison. (**E**) Association of Smc6-FLAG to a region spanning 100–200 kb from the left telomere of chromosome 7 as determined by ChIP-seq. *top2-4* cells were taken through a synchronous S-phase at 35°C, arrested in G2/M, and samples were prepared 1 hour after a temperature down-shift to 23°C (upper panel), or after 1 hour at maintained, high temperature (lower panel).

So far, the data presented here show that Smc5/6 complex is recruited to a chromosome structure which requires sister chromatids that are held together by cohesin. It also accumulates at cohesin sites along chromosome arms after replication under Top2-inhibting conditions. The structure is not a recombination intermediate, a DNA break, nor a replication fork. Neither does it appear after inactivation of Top2 in G1- or G2/M-arrested cells. Altogether this points to that the chromosomal association of Smc5/6 indicates the presence of a recombination-independent structure, which forms during replication on cohesed sister chromatids, and normally is removed by Top2. To test if Smc5/6 accumulation on chromosomes in *top2-4* was sensitive to Top2 activity after the completion of DNA replication, the chromosomal association of Smc6 was investigated after restoration of Top2 function in G2/M-phase. Mutant *top2-4* cells were first taken through an S-phase at restrictive temperature, and when arrested in G2/M, the temperature was decreased to permissive during 1 hour before sample preparation. Cell survival experiments suggest that SCIs are removed under these conditions [48], and we confirmed this by showing that the temperature down-shift rescues the segregation of chromosome 5, and removes the accumulation of SCIs on a reporter plasmid (Figure 10A–C). Under these conditions, ChIP-seq and ChIP-qPCR showed that Smc6 dissociates from chromosomes in *top2-4* cells to levels similar to those found in wild-type (Figure 10B, D and E). If the temperature instead is maintained during the prolonged G2/M-arrest, Smc6 levels remained high. This indicates that the chromosomal association of Smc5/6 correlates with a segregation-inhibiting structure that can be removed by Top2 after the completion of replication, but persist during a prolonged G2/M-arrest when Top2 is non-functional.

### Levels of chromosome-bound Smc5/6 predict the degree of missegregation in *top2-4* cells

**Figure 11:**
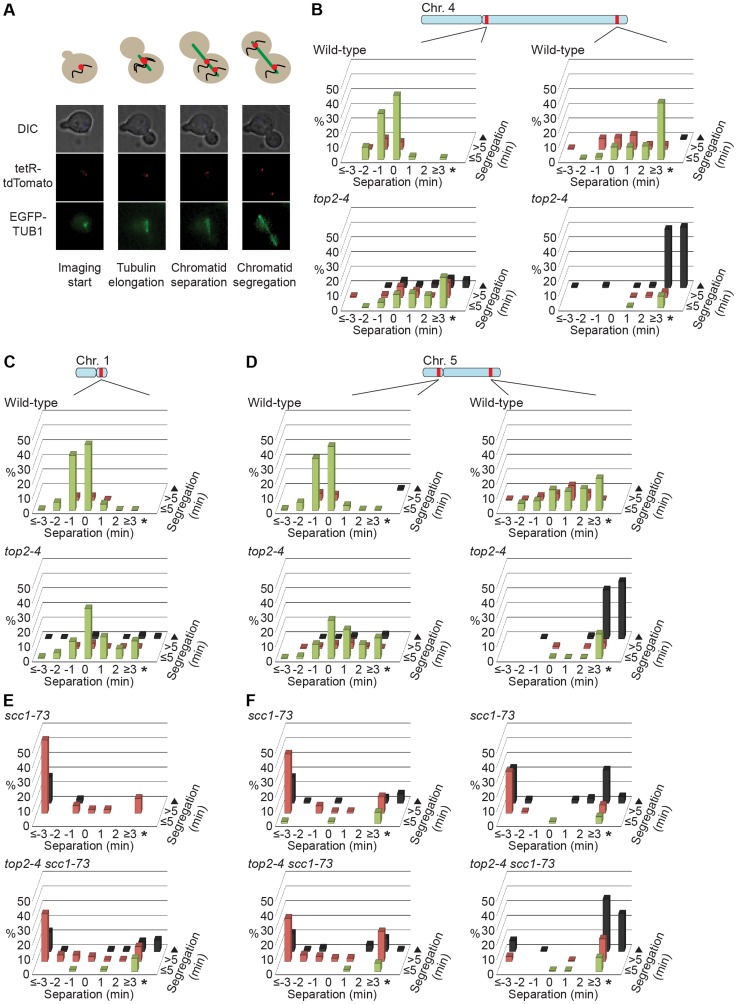
Chromosome segregation in wild-type, *top2-4*, *scc1-73* and *top2-4 scc1-73* cells. (**A**) Representation of experimental setup. Wild-type, *top2-4*, *scc1-73*, or *top2-4 scc1-73* cells harboring multiple copies of tetracycline operators at a specific chromosomal region, and expressing GFP-tagged tubulin and tdTomato-marked tetracycline repressors, were imaged during progression through one synchronous cell cycle under conditions which inactivated both mutant alleles. Elongation of the tubulin spindle was used as time point zero (0). Chromatid separation was defined as the moment when the tdTomato signal was split into two, segregation when each of the chromatid-marking dots was found in mother and bud cell, respectively. On the X-axis, bars at time point 0 represent the sum of separation events in the images collected at 0 and 0.5 minutes, time point 1 minute the sum of separation events in the frames of 1 and 1.5 minutes etc. Cells in which chromatids do not separate during the 70 minutes of imaging fall in into the category of events which are marked with an asterisk on the X-axis. If segregation occurs within 5 minutes of separation, the cells fall into the “green-bar-category” on the Z-axis (≤5 minutes). If segregation occurs more than 5 minutes after separation, the cells fall into the “red-bar-category” on the Z-axis (>5 minutes). Cells that do not segregate their chromatids during the entire 70 minutes of imaging are placed into the “black-bar-category” on the Z-axis (triangle). (**B-D**) Separation and segregation of chromosomes 4 (**B**), 1 (**C**), 5 (**D**) in wild-type and *top2-4* cells. (**E** and **F**) Separation and segregation of chromosomes 1 (**E**) and 5 (**F**) in *scc1-73* and *top2-4 scc1-73* cells. The tetracycline operators are integrated 35 kb from the centromeres on chromosomes 1, 5 and 4. This places these markers at 44, 117 and 1045 kb away from the telomeres. On chromosomes 5 and 4 the telomere proximal sites are placed 350 and 995 kb away from centromere, respectively. This places them at 72 and 85 kb away from respective telomeres.

To investigate the correlation of the chromosomal association of Smc5/6 and missegregation in *top2-4* further, we analyzed chromosome segregation after inactivation of Top2 in G2/M. Under these conditions Smc5/6 chromosomal association remains at wild-type levels (Figure 6), in contrast to the accumulation of Smc5/6 on chromosomes when Top2 is inactivated from G1 until G2/M (Figure 5). This allows segregation analysis under Top2-inhibiting conditions of chromosomes with either wild-type or increased levels of Smc5/6. In preparation for this analysis, we first investigated chromosome segregation in wild-type and *top2-4* cells released from a G1-arrest into restrictive conditions for the mutant. Using a system based on the association of fluorescently labeled tetracycline repressors with multiple repeats of tetracycline operators [9], the centromere- and telomere-proximal regions of a short (chromosome 1), an intermediate (chromosome 5) and a long chromosome (chromosome 4) were observed (see material and methods for details) (Figure 11). All three chromosomes were marked 35 kb away from the centromere and within 100 kb from one of the telomeres. Note that on the short chromosome 1, the centromere and telomere marker is one and the same. To get as detailed a picture of the segregation event as possible, sister chromatid separation was scored in relation to elongation of the mitotic spindle, and segregation was scored in relation to the separation (Figure 11A). Chromatids were logged as separated as soon as two fully separated fluorescent dots were visible, and noted as segregated when these dots were partitioned into mother cell and bud. Both separation and segregation occurred simultaneously at the centromere of all three chromosomes in wild-type cells (Figure 11B–D). Separation and segregation of the telomere-proximal regions of chromosomes 4 and 5 took place later, with the longer being most delayed (Figure 11B–D). This result is expected since segregation starts at the centromeres due to their attachment to the mitotic spindle. In *top2-4*, chromatid separation and segregation proceeded slower in the pericentromeric regions of all three chromosomes, and the delay was most pronounced on the longest chromosome 4 (Figure 11B–D). Both separation and segregation of the telomere-proximal region of chromosomes 4 and 5, but not 1, were severely impaired. These length-dependent delays are in accordance with the observation that long linear chromosomes break more frequently than short ones in *top2-4* cells, which was suggested to reflect the ability of SCIs to swivel off the ends of shorter chromosomes [6].

**Figure 12:**
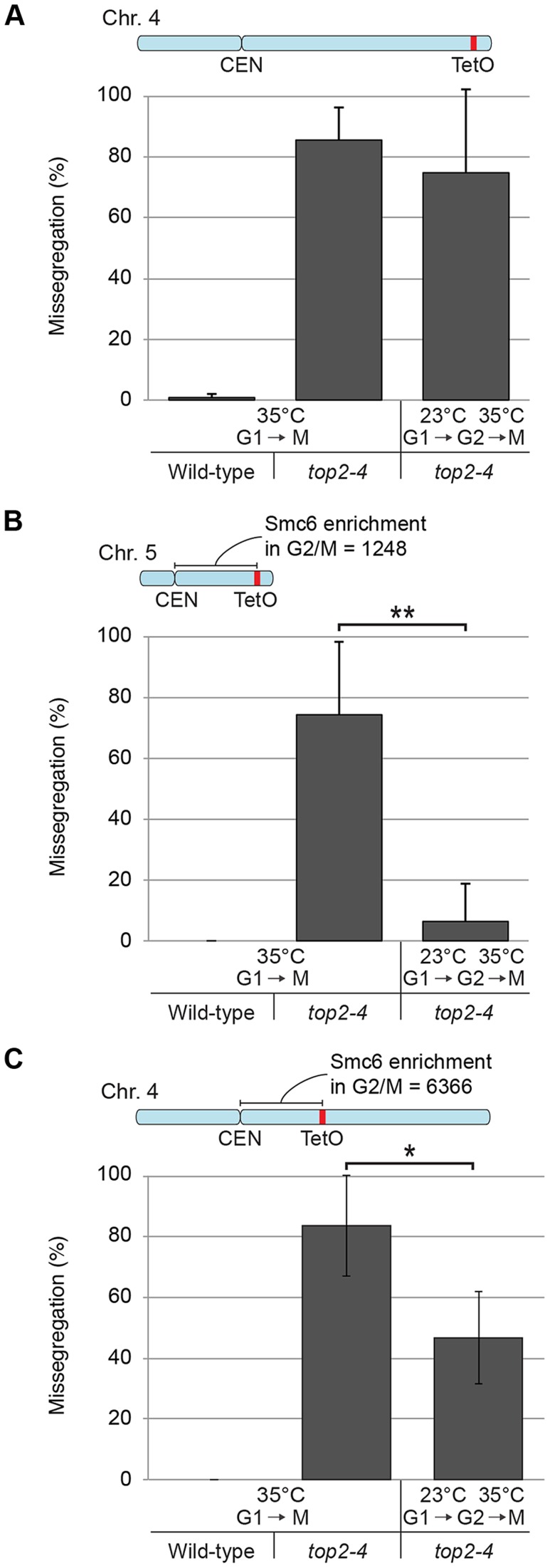
Chromosome-bound Smc5/6 predicts the degree of missegregation after Top2 inactivation. (**A**–**C**) Segregation of a fluorescently labeled region located ∼995 kb from the centromere of chromosome 4 (**A**), and ∼350 kb from centromere of chromosome 4 (**B**), and 5 (**C**), in wild-type and *top2-4* cells. The level of Smc6 enrichment in the 350 kb region between the centromere and the tetracycline operators on chromosome 4 and 5, calculated as in Figure 4, is indicated in the schematic maps of the chromosomes above each panel. Cells were first arrested in G1 at permissive temperature for *top2-4* (23°C), and thereafter either incubated at the restrictive temperature 35°C during 30 minutes before release at 35°C, or released into a G2/M-arrest at 23°C. In the G2/M-arrest the temperature was raised to 35°C for 1 hour before release at the high temperature. Segregation was subsequently scored as in (Figure 11A) in both cell populations.

Having established this, segregation was scored after inactivation of Top2 in G2/M. Again, since chromosome-bound Smc5/6 is maintained at wild-type levels under these conditions, the segregation defects should be less severe than after Top2 inactivation in G1, if the complex indicates the presence of the chromosome segregation-inhibiting structures in *top2-4* cells. Moreover, a shorter chromosome is expected to segregate more efficiently than a longer one. We therefore analyzed segregation of telomeric markers on chromosome 4 (long) and 5 (intermediate) after a shift to restrictive conditions for the *top2-4* allele in G2/M-arrested cells. This showed that in sharp contrast to the severe segregation defect of both chromosomes when Top2 is inactivated in G1, the partitioning of the intermediate-size chromosome 5 now occurred at close to wild-type levels, while the telomeric marker on the long chromosome 4 still exhibited severely defective segregation (Figure 12A and B). When the experiment was repeated, analyzing a region on chromosome 4 which was located at the same distance from the centromere as the telomeric marker on chromosome 5 (approximately 350 kb from the centromere), an intermediate improvement of segregation was detected, as compared to when Top2 was inactivated in G1 (Figure 12C).

In yet another test of how well Smc5/6 chromosome association correlates with missegregation after Top2 inactivation, segregation was scored in an *scc1-73 top2-4* double mutant after a G1-release into restrictive conditions. This analysis was also prompted by the observation that entanglements remains between circular mini-chromosomes in the double mutant [25]. Well in line with the ChIP and IF analyses, which indicate that some, but not all, Smc5/6 on chromosomes is retained but de-localized in *scc1-73 top2-4* (Figures 5A, C, D and 2D), these cells displayed an intermediate missegregation phenotype (Figure 11E and F). While centromere-proximal regions of chromosomes 1 and 5 displayed premature separation similar to the *scc1-73* single mutant, the telomere proximal site of chromosome 5 was inhibited as in *top2-4* cells.

### Smc5/6 facilitates segregation of short chromosomes when Top2 is inhibited

**Figure 13:**
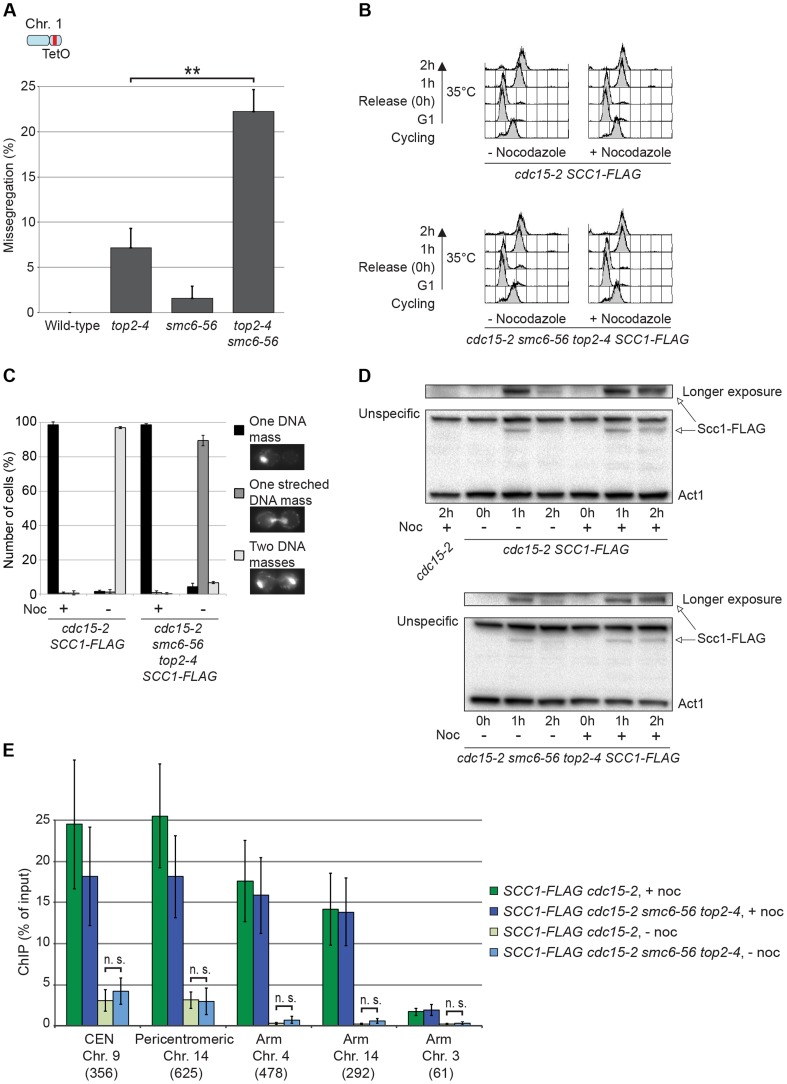
Smc5/6 promotes segregation of intertwined chromatids without perturbing cohesin removal. (**A**) Segregation of a fluorescently labeled region located 35 kb away from the centromere of chromosome 1 in indicated strains. Cells were first arrested in G1 at 23°C, and then incubated at 35°C for 30 minutes before release at maintained temperature. Segregation was scored during following anaphase as in Figure 11A. (**B**) FACS analysis of *cdc15-2 SCC1-FLAG* and *cdc15-2 smc6-56 top2-4 SCC1-FLAG* cells. Cells were first arrested in G1 at 23°C, and then incubated at 35°C for 30 minutes before release at maintained temperature in the absence or presence of nocodazole. Samples for analysis of nuclear division, Scc1 proteins levels in solution and on chromatin were collected 2 hours after release. (**C**) Nuclear division 2 hours after release. Cells were fixed in 3,7% formaldehyde for 10 minutes and stained with DAPI and analyzed under microscope. Cells were scored into either of the following three categories; one DNA mass, one stretched DNA mass bridging the mother cell and daughter bud or two separated DNA masses, one in the mother cell and one in the daughter bud. Cells released in the presence of nocodazole showed no sign of nuclear division. In contrast, *cdc15-2 SCC1-FLAG* cells released in the absence of nocodazole had undergone anaphase and were arrested in telophase with two DNA masses. *cdc15-2 smc6-56 top2-4 SCC1-FLAG* cells released in the absence of nocodazole had commenced anaphase but failed to separate the DNA into two masses, displaying one stretched DNA mass. (**D**) Scc1 protein levels detected by western blot using an anti-FLAG antibody. Actin was used as loading control. (**E**) Scc1-FLAG chromosome association as determined by ChIP-qPCR. Note that both total levels and chromosome associated Scc1 are similarly reduced in the two strains when arrested in a *cdc15-2*-induced telophase arrest (without nocodazole).

If Smc5/6 accumulates in response to the accumulation of segregation-inhibiting structures in *top2-4* mutants, it is expected to execute a function at these sites. To test this, we analyzed segregation of chromosome 1. This chromosome segregates at near to wild-type levels in *top2-4* cells despite the occurrence of new Smc6 binding sites along the arm (Figure 11C). In an *smc6-56 top2-4* double mutant however, there was a threefold increase in missegregation (Figure 13A). This indicates that the additional Smc5/6 complexes recruited upon Top2 inhibition facilitate resolution of this chromosome. To test if this function is to promote removal of cohesin from mitotic chromosomes, levels of FLAG-tagged Scc1 was analyzed by western blot and ChIP-qPCR. This showed that both total levels and chromosome-associated Scc1 was equally reduced in telophase-arrested *smc6-56 top2-4* and wild-type cells (Figure 13B–E).

## Discussion

This investigation was launched to understand why Smc5/6 accumulates on chromosomes under Top2-inhibiting conditions. Based on the current knowledge of both the complex and the topoisomerase this could either be due to the accumulation of SCIs or an increased level of sister chromatid recombination structures. Since Top2 impairment also delays replication termination, there is also a possibility that the accumulation of Smc5/6 is due to remaining forks in the mutant [45]. If recombination and/or SCI are the triggers, a central feature for Smc5/6 chromosome association should be a dependency on the proximity of sister chromatids. Using high-resolution ChIP-seq, ChIP-qPCR and IF in combination with a variety of mutations which disrupt sister chromatid cohesion, we show that this is the case in both wild-type and *top2-4* cells (Figures 2 and 5). While it was already established that the cohesin loader Scc2 is needed for Smc5/6 chromosomal association [28], the role of cohesin was more uncertain, making it possible that Scc2 directly loaded Smc5/6 on to chromosomes. However, the here presented results indicate that the absence of chromosome-bound Smc5/6 in *scc2-4* cells is due to the lack of cohesion, and not to a direct role of Scc2 in Smc5/6 loading (Figure 3). On a more general level, the results also argue that phenotypes of mutations which disrupt cohesin function are caused by the combined loss of chromosome-bound cohesin *and* Smc5/6. Mutations that change the localization of cohesin might also influence where Smc5/6 is found on chromosomes. Possibly, Smc5/6 contributes to some of the many functions assigned to cohesin (reviewed in [49]). Importantly, however, while cohesin impairment leads to cohesion loss, inhibition of Smc5/6 only creates minor cohesion defects [50], [51], with replication delays and/or perturbations of chromosome structure and segregation being more common phenotypes. This suggests that the complex regulates a process and/or structure which is specific for tethered sister chromatid pairs.

In addition to reveal that the chromosomal association of Smc5/6 in *top2-4* cells is dependent on cohesion (Figure 5), this study shows that there are no signs of unfinished duplication in the mutant at the sites where Smc5/6 accumulates (Figures 8 and 9). This argues against the possibility that Smc5/6 binding is triggered by the presence of remaining replication forks. This is further supported by the persistence of chromosome-bound Smc6 during a prolonged G2/M-arrest (Figure 10D and E), since termination of replication has been shown to be delayed but not prevented in mutants of Top2 [45]. Also, Smc6 does not accumulate on chromosomes in *rrm3Δ* cells (Figure 8C), in which fork pausing is frequent. It is also unlikely that the trigger for Smc5/6 binding in *top2-4* is a DNA break or a recombination structure since the damage checkpoint protein Rad53 remains un-phosphorylated, and the Top2-dependent increase in Smc6 binding is still present in *top2-4 mre11Δ* and *top2-4 rad52* cells (Figure 7). Moreover, there are no signs of recombination intermediates detected by two-dimensional gel electrophoresis in the DNA regions bound by Smc5/6 in *top2-4* mutants (Figure 9).

This leaves SCIs as the most likely candidates as triggers for Smc5/6 binding, and the following results argue in favor for this assumption. First, as stated above, the buildup of Smc5/6 in *top2-4* cells requires the proximity of chromatids. Second, the accumulation requires that the mutant pass through S-phase under restrictive conditions. After inactivation of Top2 in G1- or G2/M-arrested cells, the levels of Smc5/6 binding are unchanged, i. e. under conditions when Top2 inhibition is expected to perturb transcription only (Figure 6). Third, Smc5/6 dissociates from chromosomes when Top2 function is restored after replication, under conditions when Top2 resolves SCIs (Figure 10). Fourth, the level of Smc5/6 chromosome enrichment correlates to the degree of missegregation in *top2-4* cells (Figures 4 and 11). Moreover, inactivation of Top2 in G2/M, which leaves the amount of Smc5/6 binding at wild-type levels, also leads to a lower degree of missegregation than after an S-phase without Top2 function (Figure 12). In addition to this, the observation that Smc5/6 is needed for segregation of short chromosomes in *top2-4* cells (Figure 13), reveals yet another functional connection between Smc5/6 and SCIs. Results from our earlier analysis suggest that Smc5/6 facilitates formation of SCIs during replication, at least in *top2-4* cells. This function was attributed a role of the complex in facilitating fork rotation, thereby decreasing the level of replication-induced supercoiling [26]. The here presented data suggests that Smc5/6 also is needed for Top2-independent resolution of SCIs when replication has been completed (see below). Whether the replicative and post-replicative functions are functionally connected remains to be determined.

In addition to providing evidence for Smc5/6 being controlled by the presence of SCIs on chromatids, the level of its chromosomal association indicates that it senses replication-induced superhelical tension. It is difficult to envisage another mechanism that would lead to a correlation between levels of chromosome-bound Smc5/6 and the length of the shortest chromosome arm (Figure 4H). In a previous investigation, we proposed that the link between Smc5/6 binding and chromosome length reflected the ability of SCIs to swivel off chromosome ends [26]. But the relatively poor correlation between Smc6 enrichment and the length of each chromosome arm detected in this investigation (Figure 4G) argues against this, since SCI movements are expected to be confined between the microtubule-attached kinetochore and each telomere. We propose instead that the chromosomal association of Smc5/6 reflects the dissolution of replication-induced superhelical stress through rotation of the shortest arm. Such unidirectional dissolution should be possible since kinetochores become unattached from the mitotic spindle during their replication in early S-phase [39], [52], [53]. With increasing length of the shortest arm, the more difficult it will be to rotate, which will lead to higher levels of superhelical stress around the centromere. In addition to this, the chromosomal localization of Smc5/6 has to be promoted by a centromere specific-factor since superhelical tension is expected to reach high levels at centrally located, non-centromeric, regions of chromosomes as well. The specific maintenance of Smc5/6 close to the centromeres after Top2 reactivation in G2/M (Figure 10D) argues that this factor works by preventing Smc5/6 dissociation.

**Figure 14:**
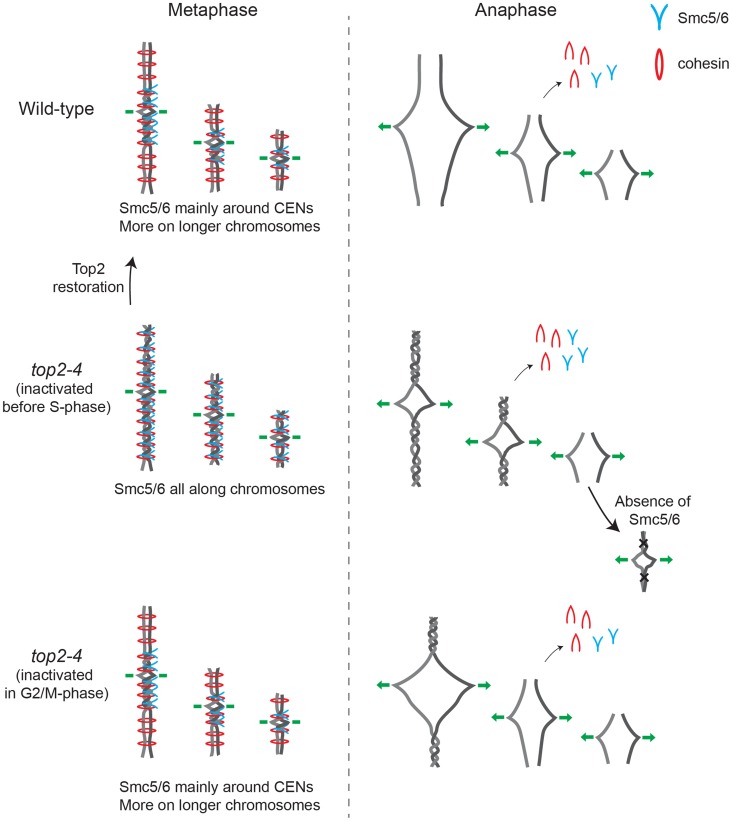
Model describing the connection between chromosome segregation and the chromosomal association of Smc5/6 and cohesin. Summary of how the chromosomal association of Smc5/6 in metaphase (*left*) correlates with chromosome segregation in anaphase (*right*) in wild-type and *top2-4* cells, in which *top2-4* is inactivated either prior or after DNA replication. See discussion for detailed description.

Taken together, the presented results are consistent with a scenario where chromosome-bound Smc5/6 indicates the presence of SCIs in the duplicated genome. Based on the observations that cohesin protects SCIs from Top2-resolution, and that Smc5/6 facilitates their resolution, it is conceivable that SCIs are positioned at Smc5/6-containing cohesin sites. Even though this cannot be formally proven until SCIs are directly observed at these sites, we use the following sections to speculate based on this model and discuss what the distribution of the complex, taken into the context of chromosome segregation in wild-type and *top2-4* cells, suggests about SCI dynamics in budding yeast (summarized in Figure 14).

Smc5/6 distribution indicates that SCIs are preferentially found in the vicinity of centromeres in wild-type cells, and accumulate along chromosome arms when Top2 is inactivated during replication (Figures 14 and S3). During chromosome segregation in wild-type cells, the pericentromeric SCIs are removed by Top2, which gain access to its substrates after proteolytic cleavage of cohesin. When Top2 is inactivated from G1 and onwards, SCIs accumulate also along chromosome arms and persist after cohesin cleavage in anaphase. The specific inhibition of segregation of intermediate and long chromosome arms under these conditions suggests that the pulling forces of the mitotic spindle drive SCIs from the centromere towards the ends of the chromosome. This will allow separation of all pericentromeric regions, and passive, Top2-independent, separation of short chromosome arms. If Top2 instead is rendered non-functional in G2/M, only centromere-proximal SCIs remain after cohesin removal, and this lower level of SCIs allows segregation of intermediate-sized chromosomes, and partial separation of central regions of a longer ones (Figure 14). Importantly, based on our observation that missegregation of the short chromosome 1 is increased in the *smc6-56 top2-4* mutant as compared to both singles (Figure 13A), Top2-independent SCI resolution appears to be facilitated by Smc5/6 function. Whether the complex achieves this by actively promoting SCI resolution via a separate mechanism and/or by preventing SCIs to be transformed into a structure which cannot be passively resolved over chromosome ends, remains to be established. However, in contrast to *S. pombe*, Smc5/6 does not appear to facilitate chromosome segregation in the absence of fully functional Top2 by promoting cohesin removal from mitotic chromosomes in *S. cerevisiae* (Figure 13D and E). This difference might reflect that Top2 inhibition specifically perturbs cohesin removal which occurs independently of Scc1 cleavage [37]. Such a pathway has been reported to exist in fission, but not budding, yeast [54]. Regardless, taking the role of Smc5/6 in the resolution of late recombination intermediates into account, it is possible that recombination structures and SCIs have something in common which allows Smc5/6 to promote their resolution.

In addition to the above, the premature chromatid separation of centromere-proximal regions in *top2-4 scc1-73* (Figures 11E and F), and the reduction in Smc5/6 chromosome association (Figure 5), suggest that cohesin does more to SCI dynamics than protecting them from Top2 resolution. If this was not the case, the segregation phenotypes of the double mutant should be identical to that of *top2-4* cells, i. e. there should be a delay in segregation at all sites tested. A possible scenario is that cohesin is also needed to prevent SCI mobility along chromosome arms, leading to an even dispersal of SCIs in the *top2-4 scc1-73* mutant. Moreover, in the lack of cohesin-imposed constraint, the pulling on the chromosomes by the mitotic spindle would be able to displace SCIs from the centromere-proximal region more readily than in a wild-type background. As a result, regions in the vicinity of centromeres would separate prematurely, while chromosome arm regions on longer chromosomes would remain entangled. On the shorter chromosomes, SCIs would also be passively resolved over chromosome ends more easily. In summary, this leads to a scenario where SCIs are resolved by Top2 decatenation and passive resolution in the *scc1-73* mutant, and only by passive resolution in *top2-4 scc1-73* cells. This is supported by the IF analysis which shows that there is more Smc6 left on chromosomes in *top2-4 scc1-73* than in *scc1-73* cells (Figure 2D).

Finally, in the light of the possibility that cohesin acts as a direct protector of SCIs we see two explanations for their preferential accumulation around centromeres in wild-type cells. One possibility is that SCI protection not only depends on cohesin, but also on a centromere-specific factor, as discussed above. The observation that reactivation of Top2 in G2/M allows removal of Smc5/6 from cohesin sites along chromosome arms, but not at centromeres (Figure 10D), argues in favor for this. Another, not mutually exclusive, scenario is that SCIs only form when the topological tension reaches a certain threshold. In wild-type cells this would only occur in the vicinity of centromeres, while in *top2-4* cells, in which replication-induced topological tension accumulates due to its function in supercoil relaxation, it would also happen at certain cohesin sites along chromosome arms. If so, chromatid entanglement after Top2 inhibition might not only be caused by lack of SCI resolution as the common view predicts, but also to an increase in SCI formation.

In conclusion, this investigation reveals that cohesin and cohesion are required for the chromosomal association and localization of Smc5/6. It also provides evidence that the chromosomal localization of Smc5/6 indicates the presence of SCIs, and that the complex is needed for their Top2-independent resolution. The localization of Smc5/6 to pericentromeric regions in G2/M-arrested cells thus opens for the possibility that SCI are maintained until anaphase, and therefore could contribute to chromatid cohesion, also on linear chromosomes. Taken together with the observation that the chromosomal localization of Smc5/6 is correlated to the length of the shortest chromosome arm, this leads to the unexpected prediction that replication-induced superhelical stress can influence chromosome segregation via the formation of SCIs.

## Materials and Methods

### Yeast strains and growth

All strains are of W303 origin (*ade2-1 trp1-1 can1-100 leu2-3,112 his3-11,15 ura3-1) RAD5* with the modifications listed in Table S1. Primer sequences used for site directed gene-modifications are available upon request.

*Strains used for live cell imaging*: To integrate multiple copies of tetracycline operators at other sites than 35 kb away from centromere 5, which is the location of the endogenous *ura3-1* gene, *ura3-1* was first replaced with the *NAT* gene, which confers resistance to nourseothricin. The *ura3-1* gene was also cloned into the PFA6a-KanMX4 plasmid, which contains the kanamycin resistance gene (*KAN*). Both *ura3-1* and *KAN* were amplified by PCR using the primers listed in Table S2. The resulting constructs were used in transformations, and correct integration at the chosen genomic sites was controlled by Southern blot. Finally, the TetO plasmid (pWJ1378) containing multiple copies of tetracycline operons and *URA3*, was integrated at the *ura3-1* sites. Correct integration was again controlled by Southern blotting. If not stated otherwise, cultures were grown in YEP medium (1% yeast extract, 2% peptone, 40 µg/ml adenine) supplemented with 2% glucose as carbon source, with the exception of the live cell imaging analysis, see below. For synchronization in G1 and a following release at restrictive temperature, 3 µg/ml α factor mating pheromone (Innovagen) was added every hour for 1.5 generation times. When a complete G1-arrest was achieved, cells were incubated at the restrictive temperature for 30 minutes, unless otherwise stated. For release into a synchronous S-phase, cells were filter-washed by three volumes of pre-heated YEP medium and subsequently resuspended in fresh medium. To achieve a subsequent arrest in the following G2/M, the release medium contained 15 µg/ml nocodazole (Sigma).

### Chromatin immunoprecipitation

Chromatin immunoprecipitation was carried out as previously described [26], [55] with the modification that cells were lysed using a 6870 Freezer/Mill (SPEX, CertiPrep). Briefly, cells were crosslinked by 1% formaldehyde and then washed three times in ice-cold 1× TBS, before being lysed in the Freezer/Mill. Cell lysate was thawed on ice and suspended in lysis buffer. Chromatin was then sheared to a size 300–500 bp by sonication and IP reactions, with anti-FLAG antibody (F1804, Sigma) conjugated to Dynabeads Protein A (Invitrogen), were allowed to proceed over night. After washing and eluting the ChIP fraction from beads, crosslinks were reversed for input and ChIP fractions and DNA was purified. The DNA samples were then processed for sequencing (see below), qPCR or hybridization to microarrays. qPCR was performed using SYBR green (Applied Biosystems) and primers listed in Table S2 on Applied Biosystem 7000 Real-Time PCR System according to the manufacturer's instructions. For ChIP-on-chip, hybridization of ChIP and input fractions to GeneChip *S. cerevisiae* Tiling 1.0R Array (Affymetrix) was performed as described [26], [55]. BrdU-IP was performed as previously described [55] using monoclonal anti-BrdU antibody (clone Bu 20a, Dako) and Dynabeads Sheep Anti-Mouse IgG (Invitrogen).

### DNA sequencing, statistical analysis and peak annotation

DNA from ChIP and WCE fractions was further sheared to an average size of approximately 150 bp by Covaris (Woburn, MA). Samples were then prepared for sequencing according to the manufacture's standard protocol (Applied Biosystems SOLiD Library Preparation protocol) and were sequenced on Applied Biosystems SOLiD platforms (SOLiD3, 4 and 5500) to generate single-end 50 bp reads. Sequenced reads of DNA-seq were aligned to the *S. cerevisiae* genome obtained from Saccharomyces Genome Database (http://www.yeastgenome.org/) using Bowtie [56], allowing three mismatches in the first 28 bases per read and filtering reads having more than 10 reportable alignments (-n3 -m10 option). Each aligned read was extended to a predicted fragment length of 150 bp. Reads were summed in 10 bp bins along the chromosomes for ChIP and WCE, and further normalized and smoothed as previously described [57], Nakato R., et al, 2013). For the number of total and mapped reads in each sample, see Table S3. Sequence data are available at the Sequence Read Archive (http://www.ncbi.nlm.nih.gov/sra) with the accession number SRP018757. To call peaks for Smc6 and Scc1, we calculated the fold enrichment (ChIP/WCE) for each bin and identified bins which fulfilled following criteria: (1) fold enrichment was more than 2.0; (2) the maximum read intensity in ChIP bins was more than 1; and (3) fold enrichment of no tag sample was less than 1.8. Chromosome arms (Figure 5E) were defined as the whole chromosomes excluding: 25 kb pericentromeric region spanning the centromere; subtelomeric regions (20 kb proximal to each telomere); and long terminal repeats (LTR). LTRs, defined by Saccharomyces Genome Database (http://www.yeastgenome.org/), were excluded from the upstream to the downstream open reading frame neighboring each LTR. The significance of Smc6 peaks clustering around pericentromeric regions (Figure S3) was assessed with the binomial test by assuming that the Smc6 peaks distributed to the whole genome uniformly. The enrichment values of Smc6-FLAG for each chromosome (Figure 4F–H) were calculated by summing up the difference of fold enrichment between Smc6-FLAG and a no tag control experiment in 100 kb regions spanning the centromeres of each chromosome (Figure S4). Detailed information on the sequencing results is found in Table S3.

### Detection of Smc6 on chromosome spreads by immunofluorescence

Mitotic spreads were prepared as described [58] with the exception that 5% Lipsol (Dynalab) was used as a detergent. Wild-type and mutated Smc6-3×HA-expressing cells were arrested in G2/M after a synchronous S-phase at 35° before preparation of spreads. Monoclonal rat-anti-HA (Roche) was used as the primary antibody followed by Cy3-conjugated goat-anti-rat (Invitrogen) to detect Smc6-3×HA on spreads. Each image was acquired under identical exposure conditions using a Leica microscope and 100× objective. Image analysis was carried out in Volocity (Perkin Elmer). Signals from >50 chromosome spreads were quantified using the analysis tools provided by the Volocity software (Perkin Elmer), and background staining in adjacent regions of the same size were subtracted. Box plots were made using standard statistical tools and represent all values measured between the maximum and the minimum. Statistical analysis to measure significance of differences between strains was done using a two-tailed T-test, with Welch's correction, which was used because the two populations compared had unequal variance. P-values greater than or equal to 0.05 were considered insignificant.

### Live cell imaging

If not stated otherwise, cells were grown at 23°C in synthetic medium lacking histidine and uracil supplemented with 2% glucose. For G1-release experiments, cells were first arrested by of alpha factor at a final concentration of 3 µg/ml, and moved to 35°C thirty minutes prior to release. 500 µl of cell suspension was then applied to Concanavalin A (Sigma) coated glass coverslips (∅ 12 mm), and were allowed to settle for 2 minutes. Medium was subsequently removed and 1 ml fresh medium without alpha factor was added. Cells were allowed to settle to the glass surface for another 40 minutes and were finally imaged through the following mitosis at 35°C. For G2-release experiments in Figure 10A, cells were first arrested in G1 as above, and after 30 minutes at 35°C, released into pre-warmed medium containing nocodazole at a final concentration of 15 µg/ml. Cells were then grown for one hour at 35°C and then either moved to 23°C or kept at 35°C for an additional hour prior to release from the G2/M-arrest. For experiments in Figure 6A, *top2-4* cells were arrested in G1, released and allowed to grow at 23°C for 90 minutes in medium containing nocodazole to reach a complete G2/M-arrest. The arrest was then maintained at 35°C for one hour prior to release. 500 µl of cell suspension was then put on Concanavalin A (Sigma) coated glass coverslips (∅ 12 mm) and were allowed to settle for 2 minutes. Medium was then removed and 1 ml fresh 23°C or 35°C medium was added as appropriate. Cells were allowed to settle on the glass surface for another 5 minutes and then imaged through the following mitosis at either 23°C or 35°C. For both type of experiments, images consisted of a 7-layer Z-stack, with layers 0.8 µm apart. These were collected every 30 seconds in green (GFP) and red (tdTomato) channels, for a total of 70 minutes. Control experiments using wild-type and recombination-deficient *rad52Δ* cells showed that this setup left cell cycle progression unperturbed, and is therefore unlikely to introduce any significant DNA damage. The microscope used was Deltavision Spectris (Applied Precision), and acquired images were analyzed using ImageJ (version 1.44i). Automated tracking of spindle length was performed using CellProfiler version r10997 [59]. Briefly, images were segmented for nuclei based on tetR tdTomato fluorescence and each nucleus was tracked over time. Within each nucleus, the EGFP-tubulin structure was segmented and tracked over time. Spindle elongation was considered when the EGFP-tubulin structure exceeded 10 pixels in length, which is equal to 3.18 µm.

### Plasmid purification and analysis

Cells containing the plasmid pRS316-URA3 were collected and immediately fixed in ice-cold 70% ethanol. These cells were subsequently pelleted and incubated at 37°C for 30 minutes in 400 µl buffer containing 0.5 mg/ml zymolyase (Seikagaku Biobusiness), 0.9 M sorbitol, 0.1 M EDTA (pH 8.0) and 14 mM β-mercaptoethanol (Sigma). After a second centrifugation, spheroblasts were resuspended in 400 µl of TE buffer and incubated at 65°C for 30 minutes with 90 µl of 270 mM EDTA (pH 8.0), 460 mM Tris-base and 2.3% SDS. Thereafter, 80 µl of 5 M potassium acetate was added, and samples were kept on ice during 60 minutes, subsequently centrifuged for 15 minutes at 13 000 rpm, and finally, the supernatant was collected into new tube. DNA was then precipitated using 1 ml of 100% ethanol, and resuspended in 500 µl of TE buffer. After treatment with 0.1 mg/ml RNaseI at 37°C for 30 minutes, the DNA was precipitated with 2-propanol, washed by 70% ethanol and resuspended in 50 µl of TE buffer. For nicking enzyme treatment, DNA was incubated with *Nb.Bts*I (New England Biolabs) for 2 hours at 37°C according to manufacturer's protocol. DNA samples were separated by electrophoresis in 0.8% agarose (Lonza) 0.5× TBE gel with 2.7 V/cm for 24 hours. Plasmids were detected by Southern blotting under standard conditions using radioactive probe that was generated by PCR using primer FW (GTTCCAGTTTGGAACAAGAGTC), primer BW (CATTAAGCGCGGCGGG) and pRS316 as template.

### Two-dimensional gel electrophoresis

Genomic DNA isolation to study replication intermediates was performed according to [60]. Isolation of genomic DNA with CTAB extraction to preserve X-shape structures was performed according to [47]. Digestion was performed using PstI-HF (New England Biolabs) for the loci *UBP10-MRPL19* and *MPP10-YJR003C*, and EcoRI and HindIII (Roche) for *ARS305* locus. The DNA was then precipitated by the addition of 2 volumes ethanol containing 0.5 M potassium acetate and incubated at −80°C for 30 minutes. The precipitated DNA was spun down for 15 minutes at 13 000 rpm and washed with 70% ethanol, before being resuspended in loading buffer. The first dimension gel running was run in 0.35% agarose (Melford, Molecular Biology Grade, MB1200) in 1× TBE at 1 V/cm, in room temperature for 24 hours. The second dimension gel running was run in 0.875% agarose (same as above) in 1× TBE with 0.3 µg/ml ethidium bromide at 5 V/cm, at 4°C for 8 hours, with buffer circulation from anode to cathode at 50 ml/min. Specific loci were detected by Southern blotting under standard conditions using radioactive probe that was generated by PCR using primer pairs GTTCGCCAGTCTCCGTTATT and CTGGGATACCCGAATGTGTATG for ARS305; ATGGTGAAGACATCGGCGAAGACA and AGTGGTAGAAGTGGTGGCTGAAGT for *UBP10-MRPL19*; GCTTCAGCGTATTGTAGCATTT and GCTCGTGGAACCTATCCTTATT for *MPP10-YJR003C*, with genomic DNA as template.

### Protein purification and western blot

To detect Rad53, wild-type and *top2-4* cells were G1-arrested at permissive temperature (23°C), incubated at restrictive temperature (35°C) for 30 min, before being released into 0,2M HU or 15 µg/ml nocodazole at 35°C for 75 min. Cells were then collected and protein extracted using trichloroacetic acid (TCA)-precipitation. To detect Scc1-FLAG in telophase and G2/M-arrests, cells were G1-arrested as above before being released into media with or without 15 µg/ml nocodazole at 35°C for 2 hours. To detect Smc6-FLAG and -HA in various strains, cells were prepared as in Figure 2. Cells were then collected and protein extracted a glass-bead disruption method [61]with the modifications that 1× PhosSTOP (Roche) was added to the lysis buffer and that after cell lysis, 2 µl of Benzonase nuclease (Novagen 70664) and NaCl to 200 mM final concentration was added and incubated 30 min at 4°C to promote the release of chromatin-bound proteins. Bradford assay was then used to estimate protein concentration and 20 µg of protein was loaded for each sample. For Rad53, Smc6-FLAG and Smc6-HA detection, membranes were cut after the blocking step and the lower part was incubated with anti-beta Actin antibody and the upper part of the membranes were incubated with anti-Rad53, anti-FLAG and anti-HA, respectively. To detect Scc1-FLAG, the membranes were not cut. Instead, the membranes were incubated with anti-FLAG and anti-beta Actin antibody simultaneously. The following antibodies were used for detection: anti-Rad53 (Abcam, ab104232), anti-FLAG (SIGMA, F1894), anti-HA (Roche, clone 3F10) and anti-beta Actin to detect Act1 (Abcam, ab8224).

## Supporting Information

Figure S1 Western blot analysis of Smc6-FLAG and -HA. (A) Protein levels of Smc6-FLAG and actin in indicated strains, grown as described in the legend of Figure 2. (B) As in (A) with the exception that Smc6 was tagged with the HA epitope instead of a FLAG epitope. Details on protein extraction and western blotting can be found in Materials and Methods.
(TIF)

Figure S2 ChIP-seq of Smc6-FLAG in the regions investigated by ChIP-qPCR. ChIP-seq of Smc6-FLAG in the regions investigated by ChIP-qPCR in Figures 2C, 3C, 5D, 7B, 10D and Figure S5B, shown for comparison. The upper panels show ChIP-seq maps from control experiment performed on cells lacking FLAG-tagged proteins. The other panels show ChIP-seq maps of Smc6-FLAG in indicated strains. Panel details and cell growth are described in Figure 2.
(TIF)

Figure S3 Chromosomal localization of Smc5/6 in wild-type and *top2-4* cells. The maps display the localization of Smc6-FLAG peaks along all the sixteen *S. cerevisiae* chromosomes (for peak annotation, see Material and Methods). The results are based on ChIP-seq analysis of samples collected after a synchronous S-phase at 35°C, restrictive temperature for *top2-4*. The red bars on the upper side of each chromosome show Smc6 localization in wild-type cells, while the blue bars below indicate the binding in *top2-4* cells. Note that Smc6 interaction sites cluster around centromeres in wild-type cells (p≤2.2×10^−16^, binominal test), but in addition spread along chromosome arms in the absence of functional Top2. Green bars denote the positions of the centromeres (CEN), green asterisks denote the position of the tetracycline operators used for the chromosome segregation assays and the grey bar on chromosome 12 denotes the position of the rDNA.
(TIF)

Figure S4 Smc6 enrichment in pericentromeric regions correlates with chromosome length and the distance from the centromere to the nearest telomere. ChIP-seq data used for the analysis in Figure 4F–H. Association of Smc6-FLAG in wild-type cells (upper panels) to 100 kb regions spanning each of the sixteen budding yeast centromeres. The lower panels display results from control experiment on cells lacking tagged proteins. Samples preparation and panel details are described in the legend of Figure 2.
(TIF)

Figure S5 ChIP-qPCR of Smc6-FLAG in a Top2-degron strain. (A) FACS analysis of wild-type, *top2-4*, a degron background strain and a Top2-degron strain. Wild-type and *top2-4* cells were arrested in G1 at 23°C, then the temperature was raised to 35°C for 30 minutes and released at maintained temperature. The degron background and Top2-degron strains were G1-arrested as above but 1 hour prior to release 1 mM auxin (3-Indoleacetic acid) and 5 µg/ml doxycycline was added to promote the degradation of Top2 and to repress the transcription of Top2, respectively. As above, the temperature was raised to 35°C for 30 minutes prior to release at 35°C into medium containing 1 mM auxin and 5 µg/ml doxycycline. (B) ChIP-qPCR of Smc6-FLAG in a degron background strain and in a Top2-degron. Cells were grown as in (A), with the difference that they were released from G1 into medium also containing nocodazole to induce G2/M-arrest. Sample were collected 75 minutes after release.
(TIF)

Table S1 Yeast strains used in this study. All strains are of W303 origin (*ade2-1 trp1-1 can1-100 leu2-3,112 his3-11,15 ura3-1) RAD5*, with the modifications listed below.
(DOCX)

Table S2 Primers used for ChIP-qPCR and for *ura3-1* integration.
(DOCX)

Table S3 Sequencing information.
(DOCX)
