# Supplementary figures and images for: The Chromosomal Association of the Smc5/6 Complex Depends on Cohesion and Predicts the Level of Sister Chromatid Entanglement
Source: PLoS Genet. 2014 Oct 16;10(10):e1004680. doi: 10.1371/journal.pgen.1004680 (PMC4199498; doi:10.1371/journal.pgen.1004680)

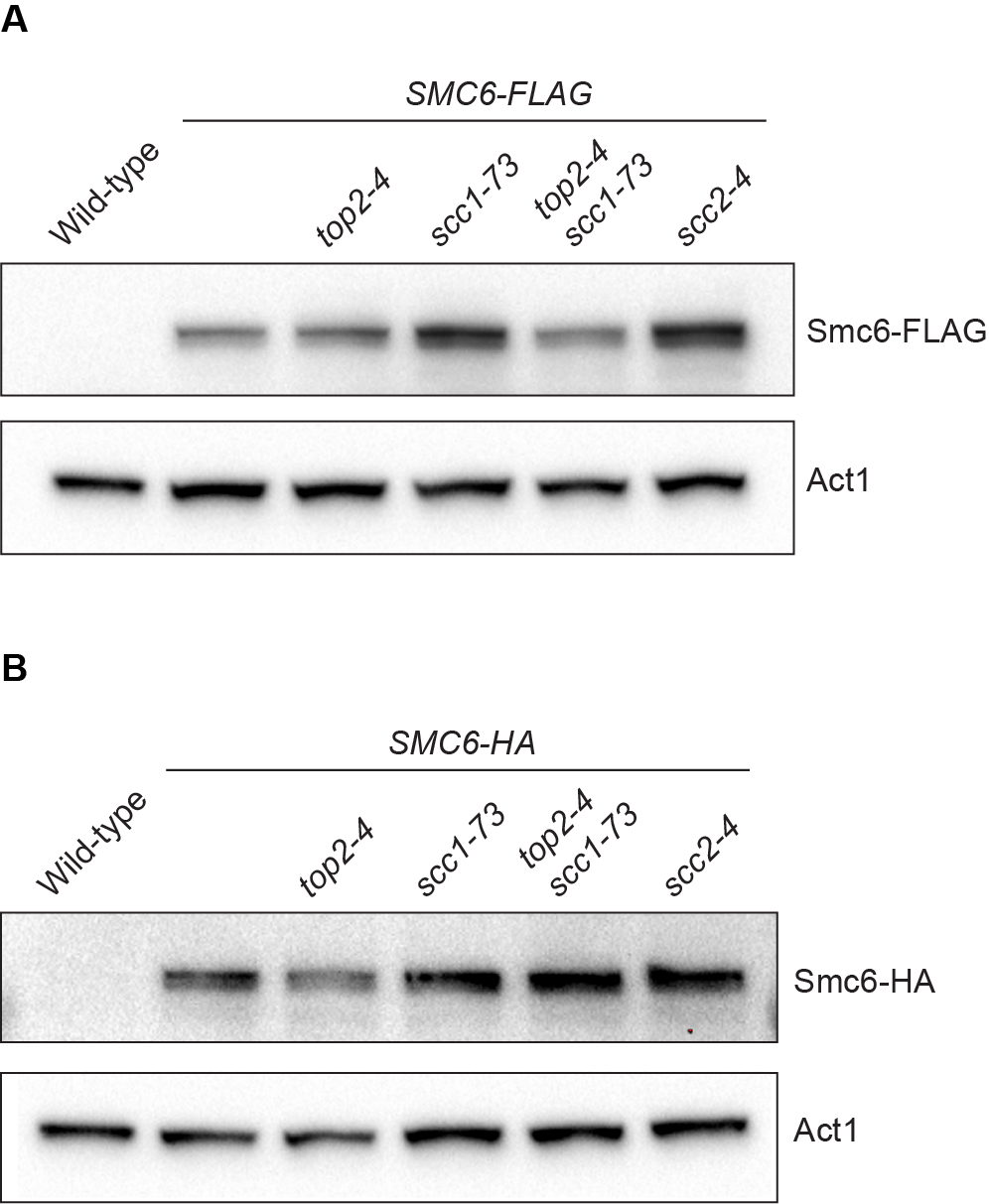

Supplement: Figure S1 — Western blot analysis of Smc6-FLAG and -HA. (A) Protein levels of Smc6-FLAG and actin in indicated strains, grown as described in the legend of Figure 2. (B) As in (A) with the exception that Smc6 was tagged with the HA epitope instead of a FLAG epitope. Details on protein extraction and western blotting can be found in Materials and Methods. (TIF) [file pgen.1004680.s001.tif]

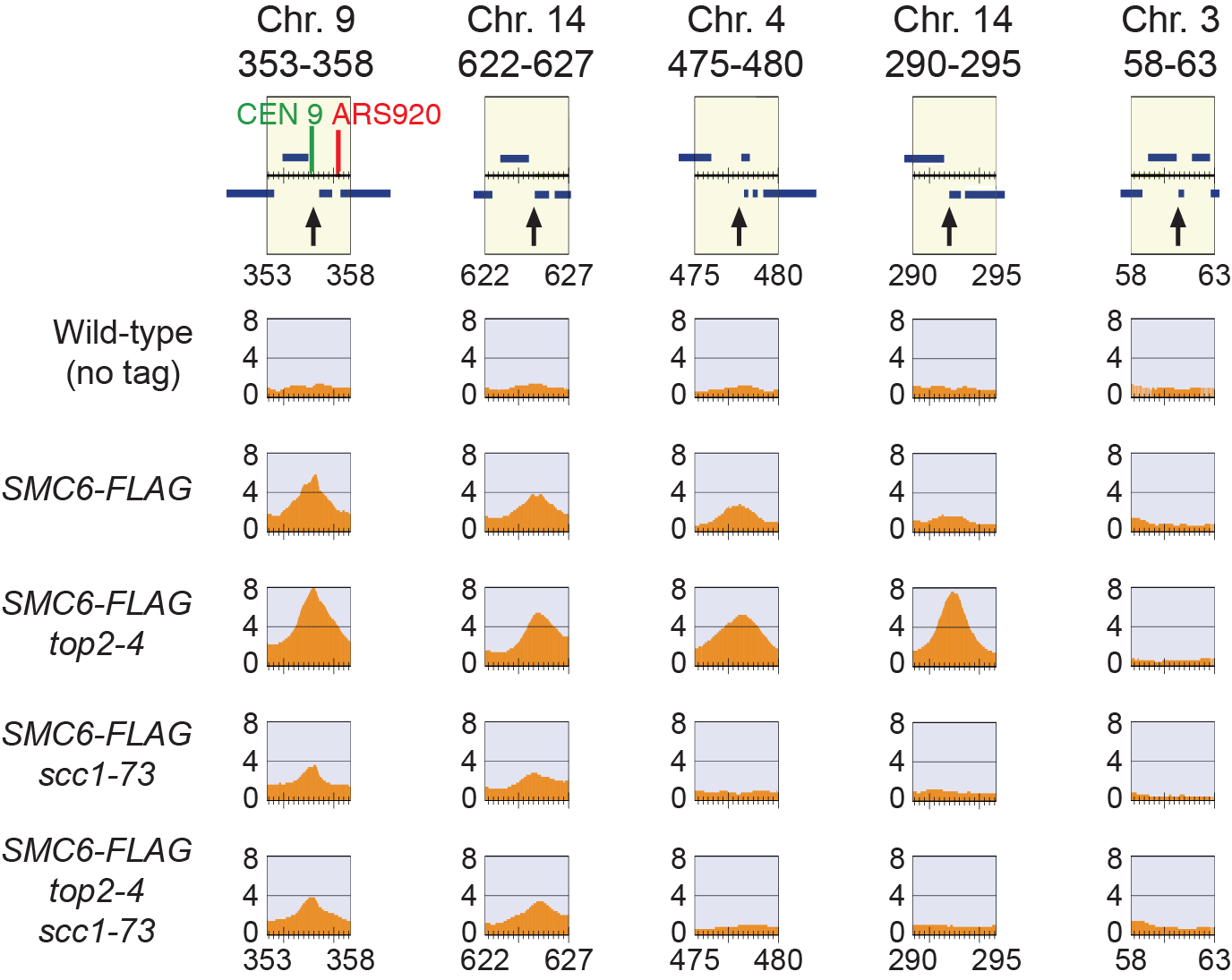

Supplement: Figure S2 — ChIP-seq of Smc6-FLAG in the regions investigated by ChIP-qPCR. ChIP-seq of Smc6-FLAG in the regions investigated by ChIP-qPCR in Figures 2C, 3C, 5D, 7B, 10D and Figure S5B, shown for comparison. The upper panels show ChIP-seq maps from control experiment performed on cells lacking FLAG-tagged proteins. The other panels show ChIP-seq maps of Smc6-FLAG in indicated strains. Panel details and cell growth are described in Figure 2. (TIF) [file pgen.1004680.s002.tif]

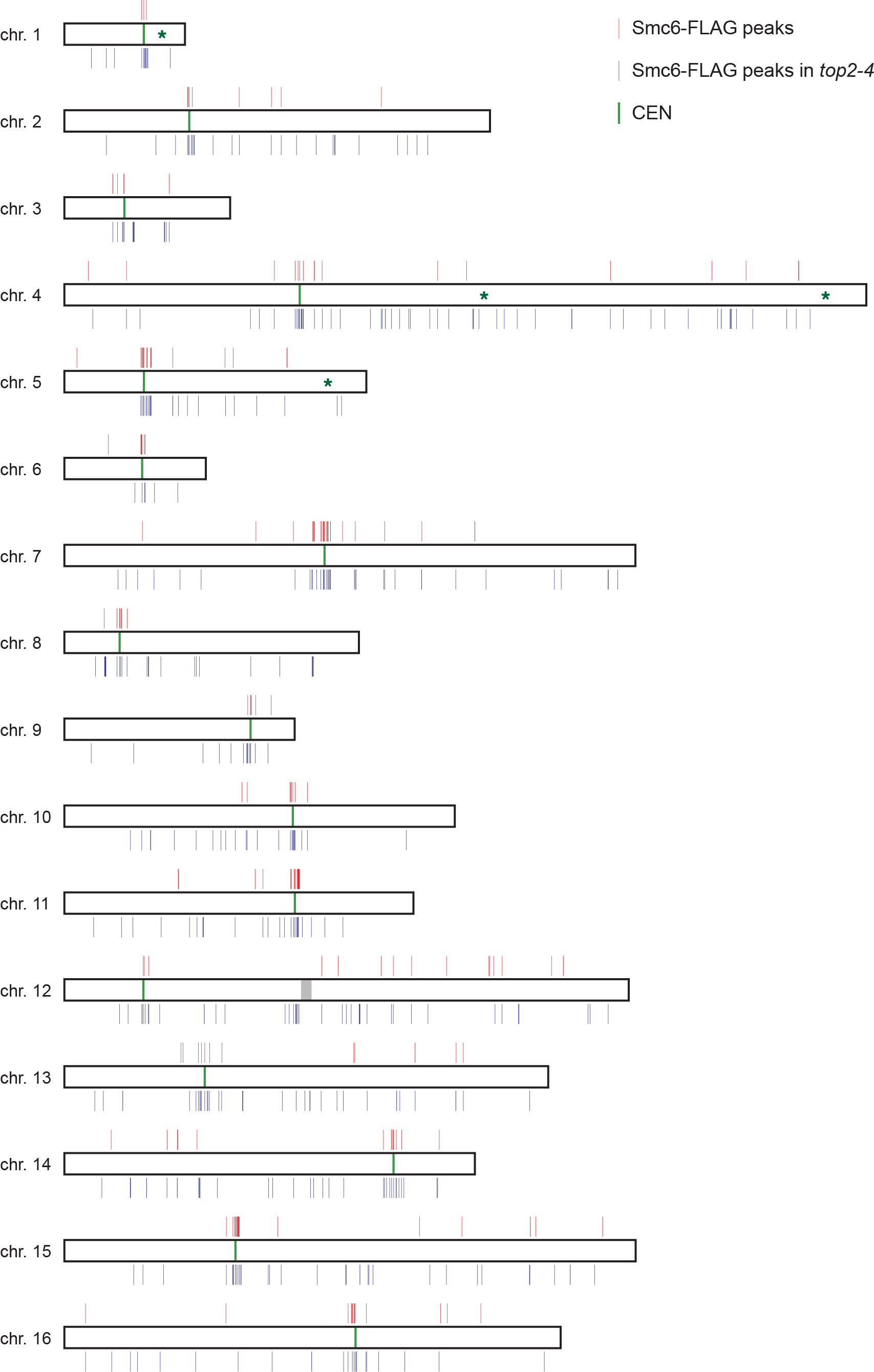

Supplement: Figure S3 — Chromosomal localization of Smc5/6 in wild-type and top2-4 cells. The maps display the localization of Smc6-FLAG peaks along all the sixteen S. cerevisiae chromosomes (for peak annotation, see Material and Methods). The results are based on ChIP-seq analysis of samples collected after a synchronous S-phase at 35°C, restrictive temperature for top2-4. The red bars on the upper side of each chromosome show Smc6 localization in wild-type cells, while the blue bars below indicate the binding in top2-4 cells. Note that Smc6 interaction sites cluster around centromeres in wild-type cells (p≤2.2×10^−16^, binominal test), but in addition spread along chromosome arms in the absence of functional Top2. Green bars denote the positions of the centromeres (CEN), green asterisks denote the position of the tetracycline operators used for the chromosome segregation assays and the grey bar on chromosome 12 denotes the position of the rDNA. (TIF) [file pgen.1004680.s003.tif]

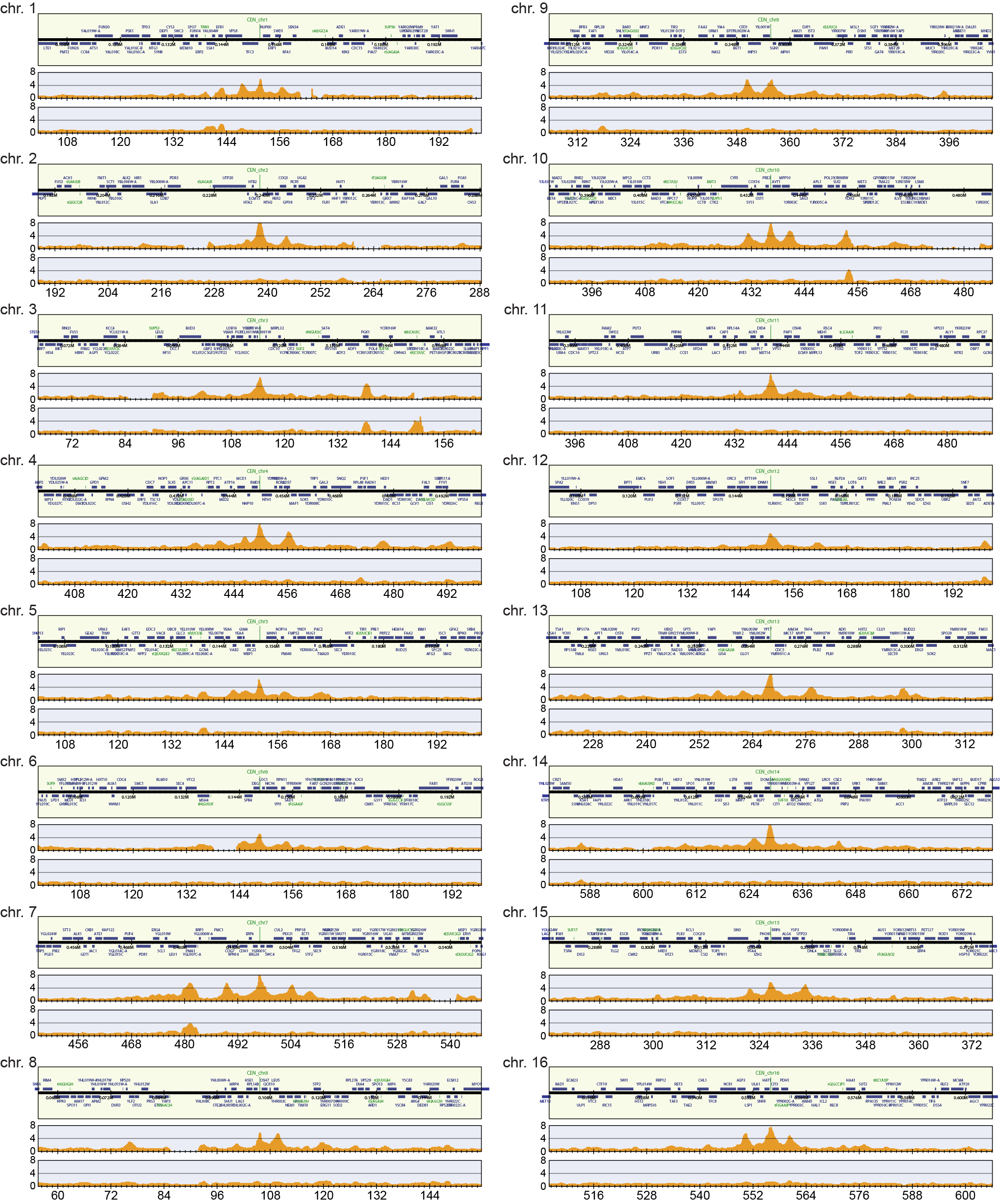

Supplement: Figure S4 — Smc6 enrichment in pericentromeric regions correlates with chromosome length and the distance from the centromere to the nearest telomere. ChIP-seq data used for the analysis in Figure 4F–H. Association of Smc6-FLAG in wild-type cells (upper panels) to 100 kb regions spanning each of the sixteen budding yeast centromeres. The lower panels display results from control experiment on cells lacking tagged proteins. Samples preparation and panel details are described in the legend of Figure 2. (TIF) [file pgen.1004680.s004.tif]

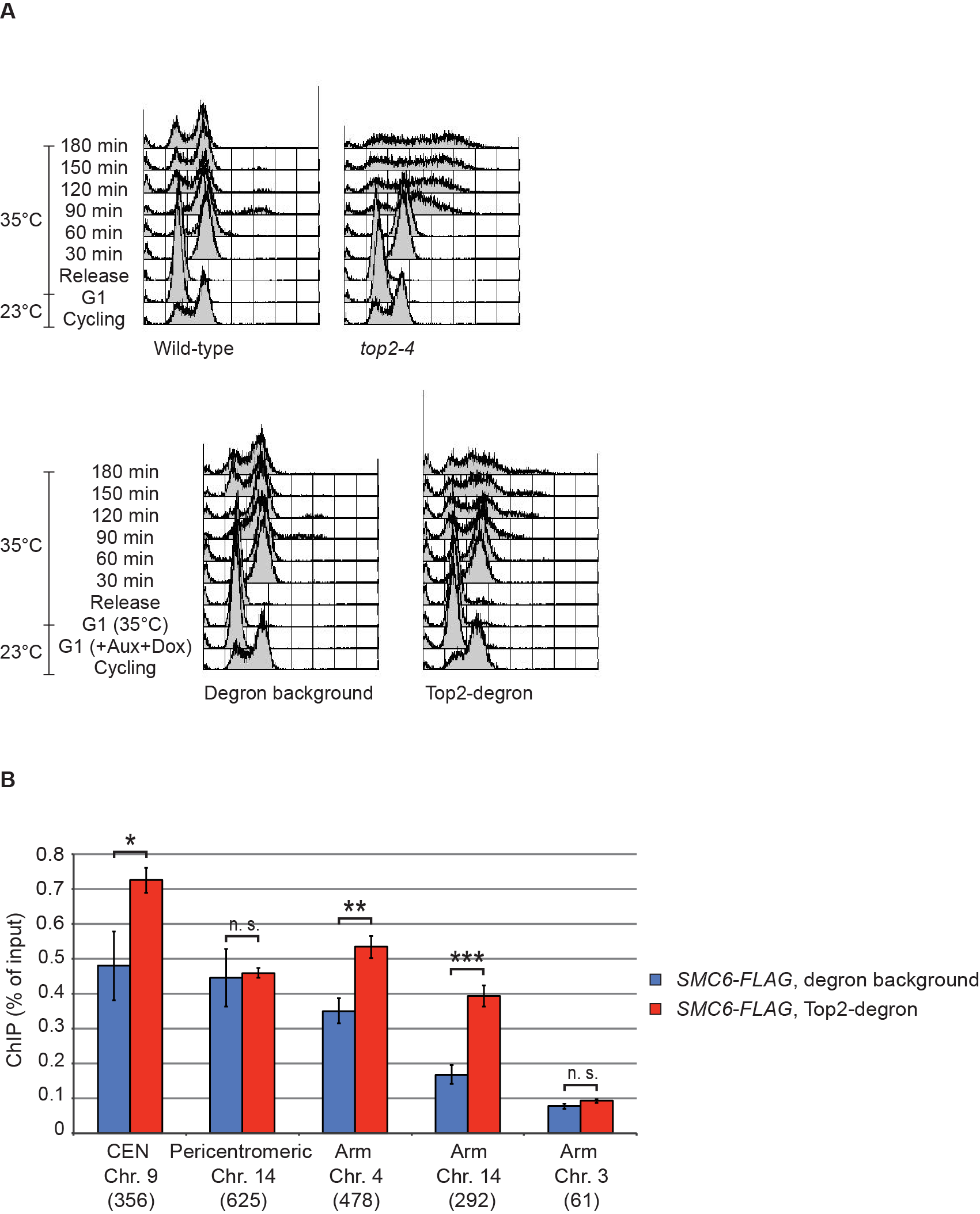

Supplement: Figure S5 — ChIP-qPCR of Smc6-FLAG in a Top2-degron strain. (A) FACS analysis of wild-type, top2-4, a degron background strain and a Top2-degron strain. Wild-type and top2-4 cells were arrested in G1 at 23°C, then the temperature was raised to 35°C for 30 minutes and released at maintained temperature. The degron background and Top2-degron strains were G1-arrested as above but 1 hour prior to release 1 mM auxin (3-Indoleacetic acid) and 5 µg/ml doxycycline was added to promote the degradation of Top2 and to repress the transcription of Top2, respectively. As above, the temperature was raised to 35°C for 30 minutes prior to release at 35°C into medium containing 1 mM auxin and 5 µg/ml doxycycline. (B) ChIP-qPCR of Smc6-FLAG in a degron background strain and in a Top2-degron. Cells were grown as in (A), with the difference that they were released from G1 into medium also containing nocodazole to induce G2/M-arrest. Sample were collected 75 minutes after release. (TIF) [file pgen.1004680.s005.tif]
