# Supplementary material for: The Chromosomal Association of the Smc5/6 Complex Depends on Cohesion and Predicts the Level of Sister Chromatid Entanglement
Source: PLoS Genet. 2014 Oct 16;10(10):e1004680. doi: 10.1371/journal.pgen.1004680 (PMC4199498; doi:10.1371/journal.pgen.1004680)
Supplement: Table S1 — Yeast strains used in this study. All strains are of W303 origin (ade2-1 trp1-1 can1-100 leu2-3,112 his3-11,15 ura3-1) RAD5, with the modifications listed below. (DOCX) [file pgen.1004680.s006.docx]

| CB67 | *MATa* |
| --- | --- |
| CB980 | *MATa top2-4* |
| CB1110 | *MATa scc1-73 top2-4 TRP1* |
| CB173 | *MATa SMC6-6HIS-3xFLAG::KAN* |
| CB1367 | *MATa top2-4 SMC6-6HIS-3xFLAG::KAN* |
| CB234 | *MATa SCC1-6HIS-3xFLAG::KAN* |
| CB383 | *MATa top2-4 SCC1-6HIS-3xFLAG::KAN* |
| CB617 | *MATa scc1-73 SMC6-6HIS-3xFLAG::KAN TRP1* |
| CB1546 | *MATa top2-4 scc1-73 SMC6-6HIS-3xFLAG::KAN TRP1* |
| CB1951 | *MATa smc6Δ::LEU2 YCplac22-smc6-56 SCC1-6HIS-3xFLAG::KAN* |
| CB1776 | *MATa scc2-4 SMC6-6HIS-3xFLAG::KAN* |
| CB2191 | *MATa pds5Δ::HIS pds5-101::LEU SMC6-6HIS-3xFLAG::KAN* |
| CB2094 | *MATa eco1-1 SMC6-6HIS-3xFLAG::KAN* |
| CB2203 | *MATa eco1-1 rad61Δ::KAN SMC6-6HIS-3xFLAG::KAN* |
| CB2189 | *MATa rad61Δ::KAN SMC6-6HIS-3xFLAG::KAN* |
| CB1533 | *MATa cdc15-2* *SMC6-6HIS-3xFLAG::KAN* |
| CB2087 | *MATa mre11Δ::HIS3 SMC6-6HIS-3xFLAG::KAN* |
| CB2003 | *MATa mre11Δ::HIS3 top2-4 SMC6-6HIS-3xFLAG::KAN* |
| CB1953 | *MATa rad52Δ::HIS3 SMC6-6HIS-3xFLAG-KAN* |
| CB1955 | *MATa rad52Δ::HIS3* *top2-4 SMC6-6HIS-3xFLAG::KAN* |
| CB1443 | *MATa DPB3-6HIS-3xFLAG::KAN* |
| CB1477 | *MATa top2-4 DPB3-6HIS-3xFLAG::KAN* |
| CB233 | *MATa NSE4-6HIS-3xFLAG::KAN* |
| CB987 | *MATa top2-4 pRS316-URA3* |
| CB1618 | *MATa ChrVCen::ura3-1::tetO::URA3 leu2-3,112::tetRtdTomato::LEU2 his3-11,15::EGFP-TUB1::HIS3* |
| CB1436 | *MATa,* *ChrIVCen::ura3-1::tetO::URA3::KAN leu2-3,112::tetRtdTomato::LEU2 ura3Δ::NAT his3-11,15::EGFP-TUB1::HIS3* |
| CB1649 | *MATa,* *ChrICen::ura3-1::tetO::URA3::KAN leu2-3,112::tetRtdTomato::LEU2 ura3Δ::NAT his3-11,15::EGFP-TUB1::HIS3* |
| CB1679 | *MATa,* *ChrVArm::ura3-1::tetO::URA3::KAN leu2-3,112::tetRtdTomato::LEU2 ura3Δ::NAT his3-11,15::EGFP-TUB1::HIS3* |
| CB1783 | *MATa, ChrIVTel::ura3-1::tetO::URA3::KAN leu2-3,112::tetRtdTomato::LEU2 ura3Δ::NAT his3-11,15::EGFP-TUB1::HIS3* |
| CB1611 | *MATa,* *top2-4 ChrVCen::ura3-1::tetO::URA3 leu2-3,112::tetRtdTomato::LEU2 his3-11,15::EGFP-TUB1::HIS3* |
| CB1551 | *MATa,* *top2-4* *ChrIVCen::ura3-1::tetO::URA3::KAN leu2-3,112::tetRtdTomato::LEU2 ura3Δ::NAT his3-11,15::EGFP-TUB1::HIS3* |
| CB1702 | *MATa,* *top2-4 ChrICen::ura3-1::tetO::URA3::KAN leu2-3,112::tetRtdTomato::LEU2 ura3Δ::NAT his3-11,15::EGFP-TUB1::HIS3* |
| CB1741 | *MATa,* *top2-4 ChrVArm::ura3-1::tetO::URA3::KAN leu2-3,112::tetRtdTomato::LEU2 ura3Δ::NAT his3-11,15::EGFP-TUB1::HIS3* |
| CB1878 | *MATa, top2-4 ChrIVTel::ura3-1::tetO::URA3::KAN leu2-3,112::tetRtdTomato::LEU2 ura3Δ::NAT his3-11,15::EGFP-TUB1::HIS3* |
| CB1912 | *MATa,* *scc1-73 TRP1 ChrVCen::ura3-1::tetO::URA3 leu2-3,112::tetRtdTomato::LEU2 his3-11,15::EGFP-TUB1::HIS3* |
| CB2068 | *MATa,* *smc6-56-6HIS-3xFLAG::KAN top2-4, ChrICen::ura3-1::tetO::URA3::KAN leu2-3,112::tetRtdTomato::LEU2 ura3Δ::NAT his3-11,15::EGFP-TUB1::HIS3* |
| CB2069 | *MATa,* *smc6-56-6HIS-3xFLAG::KAN ChrICen::ura3-1::tetO::URA3::KAN leu2-3,112::tetRtdTomato::LEU2 ura3Δ::NAT his3-11,15::EGFP-TUB1::HIS3* |
| CB1983 | *MATa,* *scc1-73 top2-4 TRP1 ChrVCen::ura3-1::tetO::URA3 leu2-3,112::tetRtdTomato::LEU2 his3-11,15::EGFP-TUB1::HIS3* |
| CB1914 | *MATa,* *scc1-73 TRP1 ChrICen::ura3-1::tetO::URA3::KAN leu2-3,112::tetRtdTomato::LEU2 ura3Δ::NAT his3-11,15::EGFP-TUB1::HIS3* |
| CB1948 | *MATa,* *scc1-73 top2-4 TRP1 ChrICen::ura3-1::tetO::URA3::KAN leu2-3,112::tetRtdTomato::LEU2 ura3Δ::NAT his3-11,15::EGFP-TUB1::HIS3* |
| CB1857 | *MATa,* *scc1-73 TRP1 ChrVArm::ura3-1::tetO::URA3::KAN leu2-3,112::tetRtdTomato::LEU2 ura3Δ::NAT his3-11,15::EGFP-TUB1::HIS3* |
| CB1870 | *MATa,* *scc1-73 top2-4 TRP1 ChrVArm::ura3-1::tetO::URA3::KAN leu2-3,112::tetRtdTomato::LEU2 ura3Δ::NAT his3-11,15::EGFP-TUB1::HIS3* |
| CB1816 | *MATa, top2-4 ChrIVARM::ura3-1::tetO::URA3::KAN leu2-3,112::tetRtdTomato::LEU2 ura3Δ::NAT his3-11,15::EGFP-TUB1::HIS3* |
| CB2395 | *MATa ura3-1::ADH1-OsTIR1-9MYC::URA3 leu2-3::tetR’-SSN6::LEU2 HIS3::tTA::tetO7-TOP2-IAA17::KANMX SMC6-6HIS-3xFLAG::KAN* |
| CB2398 | *MATa ura3-1::ADH1-OsTIR1-9MYC::URA3 leu2-3::tetR’-SSN6::LEU2 SMC6-6HIS-3xFLAG::KAN* |
| CB1843 | *MATa cdc15-2 SCC1-6HIS-3xFLAG::KAN* |
| CB2394 | *MATa cdc15-2 top2-4 smc6-56 SCC1-6HIS-3xFLAG::KAN* |
| CB120 | *MATa SMC6-3xHA::KAN* |
| CB2318 | *MATa scc1-73 SMC6-3xHA::KAN TRP1* |
| CB2324 | *MATa top2-4 scc1-73 SMC6-3xHA::KAN TRP1* |
| CB2327 | *MATa top2-4 SMC6-3xHA::KAN* |
| CB2329 | *MATa scc2-4 SMC6-3xHA::KAN* |
