# Supplementary material for: The Chromosomal Association of the Smc5/6 Complex Depends on Cohesion and Predicts the Level of Sister Chromatid Entanglement
Source: PLoS Genet. 2014 Oct 16;10(10):e1004680. doi: 10.1371/journal.pgen.1004680 (PMC4199498; doi:10.1371/journal.pgen.1004680)
Supplement: Table S2 — Primers used for ChIP-qPCR and for ura3-1 integration. (DOCX) [file pgen.1004680.s007.docx]

| Assay | Site of amplicon/integration | Sequence |
| --- | --- | --- |
| ChIP-qPCR | CEN  Chr. 9  355,775-355,872 | CACGAATACGAGATACAGGGTAATGAA |
|  |  | ACAGCTGAAGCTTGCCTCTGTATG |
|  | Pericentromeric  Chr. 14  624,890-624,999 | AGACAAGAGGCCTCAGAAGGCTTA |
|  |  | AACGGCTGGAGAAATGAGAGCGTA |
|  | Arm  Chr. 4  477,589-477,749 | GCGAGGTATAAATCTGCGTCGCTA |
|  |  | CAAGAATTTAACCTGGGCGGCT |
|  | Arm  Chr. 14  292,251-292,350 | CCTTCCATCTCCAATAAACTATGCC |
|  |  | GACCAACCCGTGCTTTAGGAGAGTTA |
|  | Arm  Chr. 3  60,701-60,891 | TCACAAGCACTCTTCCGACACACT |
|  |  | AGGGAGACTGGTGAATTGGAGGAA |
| *ura3-1* integration for live cell imaging (homology to pFA6a-KanMX4 in bold) | Chr. 1  ≈ 35 kb from CEN on short arm | AATAGAAAAAGAAAAAAAGGATCTCAAAAAGGGTTTGGTGTTGTAGTTAT**GATTCGGTAATCTCCGAGCA** |
|  |  | TTCCCTCAACTAAATTGGCACCATCTCCACTCAACACATCCAGTCTAATA**CGCACTTAACTTCGCATCTG** |
|  | Chr. 4  ≈ 35 kb from CEN on long arm | GTATGGTACCATTGTACCGCCTAATGACACATGAAGGTCATGAAGAGCAG**GATTCGGTAATCTCCGAGCA** |
|  |  | CCCTGACTCGTGTTGCCTCGAGTAAACGGTATGTCAAAAAGAATAGGTCC**CGCACTTAACTTCGCATCTG** |
|  | Chr. 5  ≈ 350 kb from CEN on long arm, ≈72 kb from right telomere | CTCTCATTACTTTATGGATCATCTCAGTATTTCACCGTTCGAGGAAAATCTTCTTTCGCAGGCCGAAGTC**GATTCGGTAATCTCCGAGCA** |
|  |  | CAGTAACATATCATCGAAGTAAAAGCTAAAACAAAGAAAGTTAAAAAAAATAAACGTACAACATAGAAGC**CGCACTTAACTTCGCATCTG** |
|  | Chr. 4  ≈ 995 kb from CEN on long arm, ≈ 85kb from right telomere | CCTCTTTGGTGCGGCGCTAACAATAGAAAAAAATACACACACATTAGATT**GATTCGGTAATCTCCGAGCA** |
|  |  | AACCTTAGTAGAGAACCGACCGTTACCTCTTTATTAGCCCTAGTTTGTAC**CGCACTTAACTTCGCATCTG** |
|  | Chr. 4  ≈ 350 kb from CEN on long arm, ≈ 730 kb from right telomere | GAAATGATGTAAATCCCAACTTTGAGCTTCCTTTCTCGTTGCTTTGTTTT**GATTCGGTAATCTCCGAGCA** |
|  |  | ACAGGAAAGAAGGTGAACGTTTAGTTTTTATCAAGGCGCCGAGTAAATTT**CGCACTTAACTTCGCATCTG** |
