# Supplementary material for: The Chromosomal Association of the Smc5/6 Complex Depends on Cohesion and Predicts the Level of Sister Chromatid Entanglement
Source: PLoS Genet. 2014 Oct 16;10(10):e1004680. doi: 10.1371/journal.pgen.1004680 (PMC4199498; doi:10.1371/journal.pgen.1004680)
Supplement: Table S3 — Sequencing information. (DOCX) [file pgen.1004680.s008.docx]

| **ChIP samples:** | **File name** | **WCE** | **Platform** | **Sequenced reads** | **Mapped reads (<=10 times)** |
| --- | --- | --- | --- | --- | --- |
| Smc6-FLAG WT | 038_1 | 1 | SOLiD3 | 24,628,794 | 15655411 (63.57%) |
| Smc6-FLAG top2-4 | 1367 IP | 2 | SOLiD4 | 18,128,583 | 12964391 (71.51%) |
| No tag | 038_2 | 1 | SOLiD3 | 37,479,042 | 22349790 (59.63%) |
| Scc1-FLAG WT | 234-2 IP | 3 | SOLiD5500 | 8,050,464 | 6366943 (79.09%) |
| Scc1-FLAG top2-4 | 383-2 IP | 4 | SOLiD5500 | 7,794,219 | 6127934 (78.62%) |
| Smc6-FLAG scc1-73 | 617 IP | 5 | SOLiD5500 | 7,993,348 | 6114288 (76.49%) |
| Smc6-FLAG scc1-73 top2-4 | 1546 IP | 2 | SOLiD4 | 12,490,071 | 9796639 (78.44%) |
| Scc1-FLAG smc6-56 | (31) 1951 IP | 6 | SOLiD5500 | 6,088,085 | 4569485 (75.06%) |
| Smc6-FLAG scc2-4 | 3, 1776 IP | 7 | SOLiD5500 | 7,357,053 | 5642000 (76.69%) |
| Smc6-FLAG eco1-1 | (49) 2094-K IP | 8 | SOLiD5500 | 7,979,210 | 6205857 (77.78%) |
| Smc6-FLAG mre11 | (47) 2087 IP | 9 | SOLiD5500 | 7,244,754 | 5693212 (78.58%) |
| Smc6-FLAG mre11 top2-4 | (15) 2003 IP | 9 | SOLiD5500 | 12,627,863 | 5466313 (43.29%) |
| Smc6-FLAG rad52 | (19) 1953 IP | 9 | SOLiD5500 | 6,460,258 | 5024368 (77.77%) |
| Smc6-FLAG rad52 top2-4 | (21) 1955 IP | 9 | SOLiD5500 | 7,398,102 | 5787053 (78.22%) |
| Dpb3-FLAG WT | 1443 G2 IP | 10 | SOLiD4 | 16,199,251 | 10501477 (64.83%) |
| Dpb3-FLAG top2-4 | 1477 IP | 10 | SOLiD4 | 17,247,201 | 12490920 (72.42%) |
| Smc6-FLAG top2-4, reduce to 23°C i G2/M | (1) 1367 23°C IP | 9 | SOLiD5500 | 7,583,411 | 5915403 (78.00%) |
| Smc6-FLAG top2-4, keep 35°C i G2/M | (3) 1367 35°C IP | 9 | SOLiD5500 | 4,757,327 | 3760393 (79.04%) |
| Nse4-FLAG WT | 038_3 | 1 | SOLiD3 | 29,985,127 | 18134652 (60.48%) |
| Smc6-FLAG cdc15-1 | 1533 IP | 12 | SOLiD4 | 19,188,075 | 13743721 (71.63%) |
| Smc6-FLAG WT G1 | 173 G1 IP | 13 | SOLiD4 | 18,782,704 | 10680262 (56.86%) |
| Smc6-FLAG 1h HU | 207 Smc6 HU 1h IP | 14 | SOLiD4 | 20,527,908 | 14625244 (71.25%) |
| Smc6-FLAG 2h HU | 207 Smc6 HU 2h IP | 14 | SOLiD4 | 19,543,183 | 14237540 (72.85%) |
| BrdU 1h HU | 207 BrdU HU 1h IP | 15 | SOLiD4 | 17,327,521 | 8691201 (50.16%) |
| BrdU 2h HU | 207 BrdU HU 2h IP | 15 | SOLiD4 | 14,229,877 | 8866115 (62.31%) |
| Smc6-FLAG WT, raise to 35°C i G2/M | 3, 173 IP | 16 | SOLiD5500 | 8,539,231 | 6556396 (76.78%) |
| Smc6-FLAG top2-4, raise to 35°C i G2/M | 3, 1367 IP | 17 | SOLiD5500 | 7,173,410 | 5527054 (77.05%) |
| Smc6-FLAG in pds5-101 | CB2191 | 18 | Hiseq2000 | 7,306,274 | 6831570 (93.50%) |
| Smc6-FLAG in rad61del | CB2189 | 19 | Hiseq2000 | 6,602,345 | 6216423 (94.15%) |
| Smc6-FLAG in rad61del eco1-1 | CB2203 | 20 | Hiseq2000 | 6,342,064 | 5976518 (94.24%) |
| Smc6-FLAG in WT 40min IP | 207 Smc6 40min IP | 22 | SOLiD4 | 17,163,930 | 12268443 (71.48%) |
| Smc6-FLAG in WT 60min IP | 207 Smc6 60min IP | 23 | SOLiD4 | 14,507,753 | 10813057 (74.53%) |
| BrdU in WT 40min IP | 207 BrdU 40min IP | 24 | SOLiD4 | 19,011,334 | 8611732 (45.30%) |
| BrdU in WT 60min IP | 207 BrdU 60min IP | 25 | SOLiD4 | 18,109,493 | 10319724 (56.99%) |
|  |  |  |  |  |  |
| **WCE samples:** | **File name** |  | **Platform** | **Sequenced reads** | **Mapped reads (<=10 times)** |
| 1 | 038_4 |  | SOLiD3 | 19,851,989 | 13552366 (68.27%) |
| 2 | 1367 SUP |  | SOLiD4 | 19,249,638 | 15554249 (80.80%) |
| 3 | 234-2 WCE |  | SOLiD5500 | 7,727,868 | 5999763 (77.64%) |
| 4 | 383 SUP |  | SOLiD5500 | 7,395,459 | 5736033 (77.56%) |
| 5 | 617 SUP |  | SOLiD5500 | 8,289,079 | 6457184 (77.90%) |
| 6 | (32) 1951 WCE |  | SOLiD5500 | 7,038,259 | 5432820 (77.19%) |
| 7 | 1776-2 WCE |  | SOLiD5500 | 6,138,332 | 4721062 (76.91%) |
| 8 | (50) 2094-K WCE |  | SOLiD5500 | 7,536,954 | 5738388 (76.14%) |
| 9 | (4) 1367 35°C WCE |  | SOLiD5500 | 6,248,069 | 4833837 (77.37%) |
| 10 | 1443 cycling SUP |  | SOLiD4 | 18,591,836 | 14743011 (79.30%) |
| 12 | 173 cycling SUP |  | SOLiD4 | 20,165,763 | 15451789 (76.62%) |
| 13 | 173 G1 SUP |  | SOLiD4 | 16,708,394 | 13390459 (80.14%) |
| 14 | 207 Smc6 HU 1h SUP |  | SOLiD4 | 16,658,984 | 11643481 (69.89%) |
| 15 | 207 BrdU HU 1h SUP |  | SOLiD4 | 12,339,085 | 7711143 (62.49%) |
| 16 | 3, 173 SUP |  | SOLiD5500 | 9,375,195 | 7287341 (77.73%) |
| 17 | 3, 1367 SUP |  | SOLiD5500 | 6,885,854 | 5285971 (76.77%) |
| 18 | 2191_WCE |  | Hiseq2000 | 6,624,665 | 5976990 (90.22%) |
| 19 | 2189_WCE |  | Hiseq2000 | 7,401,842 | 7008074 (94.68%) |
| 20 | 2203_WCE |  | Hiseq2000 | 6,546,159 | 6136290 (93.74%) |
| 22 | 207 Smc6 40min SUP |  | SOLiD4 | 19,095,535 | 13249842 (69.39%) |
| 23 | 207 Smc6 60min SUP |  | SOLiD4 | 18,335,998 | 12737499 (69.47%) |
| 24 | 207 BrdU 40min SUP |  | SOLiD4 | 20,377,778 | 13356392 (65.54%) |
| 25 | 207 BrdU 60min SUP |  | SOLiD4 | 23,043,121 | 12491604 (54.21%) |
